# Genomics-informed elucidation of trait-phenotype relationships and MABB approaches deliver major gene blast resistance in the aromatic rice landrace *Mushk Budji*

**DOI:** 10.3389/fgene.2025.1699333

**Published:** 2026-01-22

**Authors:** Asif Bashir Shikari, Raheel Shafeeq Khan, Noor-ul Ain, F. A. Mohiddin, Gazala Hassan Khan, Najeeb-ul-Rehman Sofi, Zahoor A. Dar, M. Ashraf Ahangar, Gowhar Ali, Aflaq Hamid Wani, Bilal A. Padder

**Affiliations:** 1 Division of Genetics and Plant Breeding, Faculty of Agriculture, Wadura Campus, Srinagar, India; 2 Mountain Research Centre for Field Crops, Khudwani, India; 3 National Seed Project, Shalimar Campus, Shalimar, India; 4 Division of Plant Pathology, Shalimar Campus, Sher-e-Kashmir University of Agricultural Sciences and Technology of Kashmir, Srinagar, India

**Keywords:** rice, landrace, *Mushk Budji*, blast, pyramiding, sequencing, genes

## Abstract

**Introduction:**

A novel set of pyramided lines for durable blast resistance was developed using marker-assisted backcross breeding (MABB) strategy in the genetic background of the aromatic landrace *Mushk Budji* (MB).

**Methods:**

Simultaneous-but-stepwise transfer of the blast resistance genes *Pi54* and *Pi9* was achieved through the integration of foreground selection (FS) and background genome recovery processes, aided by genome-wide SSR and >1500 KASP markers. MABB, whole genome re-sequencing, coupled with stringent phenotypic selection for aroma, amylose content, kernel dimensions, and cooking quality, helped minimize the linkage drag and achieve early recurrent parent genome (RPG) recovery in the inter-cross BC_2_F_2:3_ generation.

**Results:**

The two-gene lines carrying *Pi9* + *Pi54* were developed through inter-crossing corresponding near-isogenic lines (NILs) with an RPG of approximately 90%. With the help of sequencing of the derived NILs, we were able, for the first time, to establish the role of major alleles underlying rice quality and stress resilience in MB. In the process, we confirmed the retention of favorable alleles at key genetic loci, such as *BADH2* (aroma), *Wx* (amylose content), *Rc* (white pericarp), *Hd1/Hd4/Hd5* (heading date), and *COLD1/COLD6* (cold tolerance) in the derived NILs. GGE biplot analysis revealed stable performance of five advanced lines across target ecologies.

**Discussion:**

The set of NILs was useful in elucidating the phenotypic effects of 11 genes related to grain type, quality, and adaptability traits in the landrace MB. Multi-environment screening for blast resistance, at hot spot locations, in addition to artificial inoculation, validated the resistance response of the lines to both leaf and neck blast. This study demonstrates the successful integration of genomics-assisted breeding and phenotypic selection to improve a heritage rice variety for enhanced disease resistance and ecological adaptation. The development of improved MB lines represents a rare endeavor towards the area expansion and conservation of the heirloom rice.

## Introduction

A landrace is a population that naturally develops in a certain region under the influence of the regionally prevailing conditions of climate and soil management, without or with only mass selection (Zeven, 1998). The landraces have not been bred to a pre-determined level of performance (Rieger et al., 2012) and hence differ from the formally bred varieties. More than 100 local rice landraces have been documented from the Kashmir Valley (Najeeb et al., 2018) and characterized at morphological (Parray et al., 2008) and molecular levels (Shikari et al., 2021). Few of the popular landraces include *Mushk Budji*, *Kamad*, *Nun-Beoul*, *Lar-Beoul*, *Begum*, *Zagir*, *Kawkreer*, *Noor Miri*, and *Zag*. These landraces primarily belong to the short and bold-grained *japonica* and are recognized for their distinctive appearance, aroma, taste, and texture of cooked rice. Of these, *Mushk Budji* (MB) is a short-grained aromatic rice landrace grown in mid-altitudes (1,750–1,900 msl) of the Kashmir Valley and is known for its pleasant aroma and taste. However, high susceptibility to the rice blast pathogen, *Magnaporthe oryzae*, has resulted in a considerable decline in its area over the past few decades. Thus, an initiative was undertaken to incorporate genetic resistance into the background of MB. MB cultivation has received significant attention in recent years due to its high demand, competitiveness, and 4–5-fold yield advantage compared to contemporary high-yielding varieties. Its revival and scaling-up of production was reported as a success story from our group (Najeeb et al., 2018). MB carries a Geographical Indication tag, No. 758, for the Jammu and Kashmir region of India.

Incorporation of genetic resistance against rice blast has been advocated as an effective and eco-safe option (Hittalmani et al., 2000; Chen et al., 2001; Singh et al., 2011; Sharma et al., 2012) in order to combat the yield losses in rice. Many such endeavors have used marker-assisted backcross breeding (MABB) or the marker-assisted gene pyramiding approach to achieve durable host-plant resistance. In the blast system, 114 genes have been reported worldwide, of which 27 (*Pita*, *Pib*, *Pb1*, *Piz* ^*t*^ *, Pid2*, *Pii*, *Pik* ^*m*^, *Pit*, *Pid3*, *Pid3-A4*, *Pish*, *Pik*, *Pik* ^*p*^, *Pia*, *PiCO39*, *Pi1*, *Pi2*, *Pi5*, *Pi9*, *Pi21*, *Pi25*, *Pi33*, *Pi36*, *Pi37*, *P50*, *Pi54*, and *Pi65*) have been cloned (Samal et al., 2019). The genes that have been found to perform well in rice-growing regions of the Himalayas include *Pi54* (Sharma et al., 2002; Singh et al., 2012) and *Pi9* (Rathour et al., 2011; Khanna et al., 2015). We, in our previous study, incorporated *Pi54* in combination with yet another major blast resistance gene *Pita* in MB (Khan et al., 2018). *Pi54* belongs to the NBS-LRR class of genes and triggers the upregulation of β-1, 3-glucanase, β-glucosidase, PAL, polyphenol oxidase, and peroxidase in response to *M. oryzae* infection. *Pi54* has been found to localize in the cell cytoplasm, which affects *Avr-*interaction, followed by its subsequent interaction with different regulatory genes inside the nucleus. *Pi54* coded protein accumulates in mesophyll cells, vascular bundle cells, and plasmodesmata and activates signaling mechanisms in neighboring cells (Gupta et al., 2012). Pertinently, the gene *Pi9* is believed to be a very effective gene against *M. oryzae* races worldwide (Amante-Bordeos et al., 1992; Imam et al., 2014) and also in India (Variar et al., 2009; Thakur et al., 2015). Marker-assisted selection was employed for the rapid introgression of semi-dwarfing and blast resistance genes (*Pi9*) into a popular Basmati rice variety, ‘Ranbir Basmati’ of the Jammu and Kashmir region (Samal et al., 2019). The combination of *Pi54+Pi9* was reported to confer enhanced resistance toward both leaf and panicle blast. *Pi54* is believed to synergize well with other genes, such as *Pigm*, *Pi2*, and *Pi40* (Wu et al., 2019). Interestingly, *Pi54* provides moderate resistance to sheath and bacterial blight in rice as well. With this background as regards the performance of the individual genes, we therefore, aimed at pyramiding *Pi9* and *Pi54* in the same genetic background to achieve strong genetic resistance against rice blast.

The expectations to accelerate the RPG recovery through selection of RP alleles at a large number of loci were explained by Frisch et al. (1999) as a function of multiplicative action of selection intensity (i), standard deviation of RPG (σ), and correlation between the proportion of RP alleles at marker loci and the proportion of RP alleles across the whole genome (r). We previously advocated selection for easily observable (phenotypic) traits to enrich loci that cause a higher ‘r’ in the above equation (Khan et al., 2018). Overestimation of marker-based background genome recovery has been discussed (Khanna et al., 2015). So, we attempted to use both SSR/STS and uniplex KASP markers for the estimation of background genome recovery in NILs and followed this by validation of genomic loci underlying rice quality and agronomic traits through a sequencing-based approach. These approaches were followed to achieve the overarching objective of deriving improved *Mushk Budji* lines that combine genetic resistance with superior grain quality, thereby ensuring yield stability and commercial viability of this heirloom rice.

## Materials and methods

### Plant materials

*Mushk Budji*, a popular short-grained aromatic rice landrace, was used as the recurrent parent to pursue two parallel backcross programs involving the donor lines IRBL9W and DHMAS 70Q 164-1b for marker-assisted incorporation of the blast resistance genes *Pi9* and *Pi54*, respectively.

### DNA extraction and PCR amplification of SSR and STS markers

DNA of different backcross progenies was extracted from young leaf tissues using the cetyltrimethylammonium bromide (CTAB) method described by Murray and Thompson (1980). Polymerase chain reaction (PCR) was performed in a thermal cycler (TaKaRa, Shiga, Japan) using 25 ng of genomic DNA, a PCR reaction mix containing 1 μl of 10x PCR buffer (10 mM Tris, pH 8.4, 50 mM KCl, and 1.8 mM MgCl_2_), 2 mM dNTPs (Thermo Fisher Scientific Pvt. Ltd., Mumbai, India), 5 pmol each of the forward and reverse primers, and 3 U of Taq DNA polymerase (Thermo Fisher Scientific Pvt. Ltd., Mumbai, India) in a reaction volume of 10 μL. The PCR program for markers Pi9-Pro and Pi54 MAS was set as follows: initial denaturation at 94 °C for 5 min; followed by 36 cycles of denaturation at 94 °C for 30 s, annealing at 55 °C for 30 s, and extension at 72 °C for 1 min; and a final extension at 72 °C for 7 min. The PCR amplicons were resolved in a 2.5% agarose gel and visualized and processed using a gel documentation system (Bio-Rad Laboratories Inc., United States).

### Marker-assisted foreground and background selection

**FIGURE 1:**
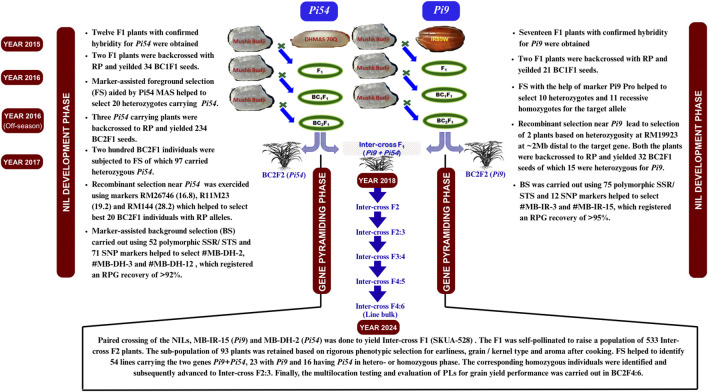
Simultaneous-but-stepwise transfer of the genes *Pi54* and *Pi9* in the genetic background of aromatic landrace *Mushk Budji.*

Simultaneous-but-stepwise MABB strategy (Singh et al., 2012) was employed to incorporate major blast resistance genes *Pi9* and *Pi54* into the genetic background of MB (Figure 1). MB was crossed as the female parent with two donors to generate F_1_ plants. From each cross, a single F_1_ plant with confirmed hybridity was backcrossed with MB through recurrent backcrossing up to BC_2_F_1_. The selected plants in BC_2_F_1_ were crossed to yield inter-cross first- and second-generation plants. Subsequently, individual plants from inter-cross generations were subjected to marker-assisted foreground selection (FS) using gene based co-dominant markers Pi9-Pro and Pi54 MAS for the genes *Pi9* and *Pi54*, respectively. The marker-assisted background selection (BS) was conducted using a combination of genome-wide SSR and KASP markers. The *Pi54*-NILs in BC_2_F_1_ were screened using 124 polymorphic SSR/KASP markers spread across the genome, with 10 markers on the carrier chromosome 11. Likewise, the *Pi9*-NILs were analyzed using 88 SSR/KASP markers, with 24 markers located on the carrier chromosome 6. Finally, the pyramided lines (PLs) carrying *Pi9+Pi54* were subjected to marker-assisted background selection using 1502 KASP (Kompetitive allele specific PCR) markers, according to the method described by Shikari et al. (2021). The extent of RPG recovery was calculated according to the method described by Khanna et al. (2015). The primers were custom-synthesized by Sigma Technologies Inc., United States. The RPG recovery was graphically represented using Graphical Geno Typing (GGT 2.0) software and Flapjack (v1.21.02.04).

### Whole genome re-sequencing and bioinformatics

A set of final NILs was selected for whole-genome sequencing using the Illumina NovaSeq X platform (Illumina, San Diego, CA, United States). Genomic DNA was isolated following a standardized protocol and subsequently normalized across samples. The extracted DNA was used to prepare paired-end sequencing libraries using the NEBNext® Ultra™ II DNA Library Prep Kit with an insert size of 350 bp, following the standard protocols provided by New England Biolabs (Massachusetts, United States) and Illumina (San Diego, CA, United States). De-multiplexing was carried out using FASTX Toolkit (version 0.0.13). FastQC (version 0.11.8, http://www.bioinformatics.babraham.ac.uk/projects/fastqc/) was used for quality check. The parameters, such as base quality score distribution, sequence quality score distribution, average base content per read, and GC distribution, in the reads were considered. Universal Illumina Adapters (AGATCGGAAGAGC) were removed using Trim Galore (version 0.6.6, https://www.bioinformatics.babraham.ac.uk/projects/trim_galore/), a wrapper script that automates quality and adapter trimming along with quality control. Oryza sativa downloaded from the Ensembl plants release 60 (https://ftp.ensemblgenomes.ebi.ac.uk/pub/plants/release-60/fasta/oryza_sativa/dna/) was used as the reference genome. Reads were mapped against the reference genome using the MEM algorithm of BWA (version 0.7.5). The key causal variant sites at the selected genes were revealed. Allelic variation for each genotype was visualized and characterized using the Integrated Genome Viewer (Robinson et al., 2011).

### Multi-location testing of pyramided lines for grain yield performance

The PLs (SKUA-528-50-1-1-3-2-1, SKUA-528-50-1-1-19-2-13, SKUA-528-50-1-1-3-2-14, SKUA-528-50-1-1-3-2-18, SKUA-528-50-1-1-19-5-3, SKUA-528-50-1-1-19-1-5, SKUA-528-50-1-1-19-6-1, SKUA-528-50-1-1-19-1-37, SKUA-528-50-1-1-19-1-94, and SKUA-528-50-1-1-3-2-8) were sown across five different locations, namely, Khudwani (E1, 1,560 m), Kulgam (E2, 1,900 m), Wadura (E3, 1,520 m), Sagam-1 (E4, 1,900 m), and Sagam-2 (E5, 1,900 m). The PLs, along with the parents, were grown in a randomized block design with two replications under irrigated ecology. Uniform management was followed at all locations under irrigated conditions. The observations were recorded on morphological and cooking quality traits: DF (days to 50% flowering), GY (grain yield per plant, g), NT (number of effective tillers per plant), SP (spikelets per panicle), SW (1000-seed weight, g), KLBC (kernel length before cooking, mm), LBR (length-to-breadth ratio), KLAC (kernel length after cooking, mm), KER (kernel elongation ratio), GC (gel consistency, mm), AC (amylose content, %), ASV (alkali spreading value), and aroma. GC was estimated following the ASV method devised by International Rice Research Institute (2013). Kernel dimensions were recorded as per our standard laboratory protocol. Aroma was tested using the panel test after properly cooking the rice samples in a water bath for 10 min. Amylose was measured via the non-destructive method using well-polished whole milled kernels (50 g). Apparent amylose content was estimated using near-infrared reflectance (NIR) spectroscopy using a Kett Grain Analyzer (Kett Electric Laboratory, Tokyo, Japan). The instrument was pre-calibrated against standard rice samples with known amylose values determined by the iodine colorimetric method, following the standard protocol of Bao and Juliano (2018). GGE biplot analysis was carried out to investigate genotype relations across different environments with respect to grain yield performance. A “which-won-where” view of the GGE biplot was constructed to characterize the genotypes based on their agronomic performance and distribution across broad environments. The biplot comprised coordinates with perpendicular equality lines drawn on its sides, which formed sectors representing specific environments. Genotypes located on the vertices of the polygon were regarded as the best performers within the sector. The AEC view of the GGE biplot, which explains genotype comparisons based on mean performance and stability across environments, was drawn to rank the genotypes on the AEC abscissa. The GGE biplot analysis was conducted using software Genstat v.12.

### Evaluation for blast disease resistance under controlled conditions

The *M. oryzae* isolates, Mo-nwi-kash-32 and SKUA-Mo-3, originating from MB and maintained at MRCFC, Khudwani, were used for screening the PLs for resistance to rice blast under controlled conditions. The seedlings were grown in 10-cm-diameter pots inside mist chambers. Inoculation was performed at the three-leaf stage by the application of 50 mL of spore suspension (∼5 × 10^4^ conidia ml^−1^), followed by incubation of seedlings for 24 h in the dark at 26 °C–27 °C. Misting was carried out every 6–7 h for 4–5 days to maintain humidity and facilitate optimal disease development. The disease was scored 7 days after inoculation using the scale described by Mackill and Bonman (1992).

### Evaluation for blast disease resistance under field conditions

The pyramids were also screened in the Uniform Blast Nursery at five hot spot locations in Jammu and Kashmir, viz., Khudwani, Kulgam, Wadura, Sagam-1, and Sagam-2. PLs were sown in a 50 × 10 cm row arrangement along with the RP and DP controls in a raised bed nursery. RP MB was sown as a spreader row after every five rows and along the borders. Disease evaluation was conducted on a 0–9 scale using the Standard Evaluation Scale of the International Rice Research Institute (2013). Lines with scores 0–3 were considered resistant, 4–5 as moderately resistant, 6–7 as moderately susceptible, and 8–9 as susceptible.

## Results

The results describe the marker-assisted development of the two-gene pyramided lines (Mushk Budji^*Pi9+Pi54*^) through development and the subsequent inter-crossing of NILs with ∼90% recurrent parent genome recovery. Sequencing and multi-environment analyses confirmed retention of key quality and adaptive alleles and identified five advanced lines showing stable performance across target ecologies.

### Marker-assisted backcross breeding

Simultaneous-but-stepwise transfer of the genes *Pi54* and *Pi9* was affected through marker-assisted foreground selection in BC_1_F_1_, BC_2_F_1_, and inter-cross F_1_ and F_2_ populations using gene-based markers Pi54 MAS and AP5659/Pi9-Pro, respectively. The selected lines were subjected to marker-assisted background selection at recombinant markers and genome wide loci.

### Pi9

#### Marker-assisted foreground selection

The recurrent parent *MB* was crossed as a female to *Pi9* donor IRBL9W to produce 38 F_1_ seeds. Seventeen heterozygous plants with early maturity were harvested, and two plants with aroma and desirable plant type were backcrossed to RP to generate BC_1_F_1_. Twenty-one BC_1_F_1_ seeds were raised, of which 10 were found to be heterozygous and 11 carried the RP allele at the Pi9-Pro marker locus. Recombinant selection was carried out near *Pi9*, and in the process, two plants were selected based on heterozygosity at RM19923, located at ∼2 Mb distal to the target gene. Two plants were backcrossed with RP to yield 32 BC_2_F_1_ seed, of which 15 were found to be heterozygous for *Pi9*.

#### Marker-assisted background selection

A polymorphism survey was carried out between RP *MB* and *Pi9* donor line IRBL9W using 290 genome-wide markers, of which 75 SSR/STS markers were found to be polymorphic between parents and were utilized to estimate the proportion of the RP genome of a set of BC_2_F_1_ lines. Furthermore, the lines were compared at 12 SNPs, which together formed 87 marker loci, including 23 background markers on carrier chromosome 6. Fifteen BC_2_F_1_ plants carrying *Pi9* registered a recovery in the range of 77.01%–96.55%. Of these, the lines MB-IR-3 and MB-IR-15 recorded RPG of 95.4% and 96.5%, respectively. These lines carried a minimum linkage drag of approximately 3 Mb near the target (*Pi9*) locus and showed the RP allele at the *Wx* gene (Supplementary Tables S1 and S2).

### Pi54

#### Marker-assisted foreground selection

**FIGURE 2:**
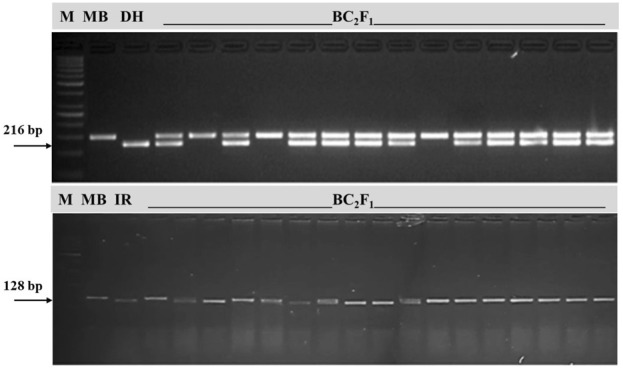
Marker-assisted foreground selection for major blast resistance genes in BC_2_F_1_. [Upper lane: *Pi54* (Pi54 MAS), Mushk Budji/DHMAS 70Q 164-1b//Mushk Budji; lower lane: *Pi9* (Pi9-Pro), Mushk Budji/IRBL 9W//Mushk Budji; MB, Mushk Budji; DH, DHMAS 70Q 164-1b; IR, IRBL9W].

In a cross between MB x DHMAS70Q 164-1b, 12 F_1_ plants were selected for heterozygosity, of which two were used for backcrossing with RP MB. Thirty-four BC_1_F_1_ plants were subjected to FS, of which 17 individuals amplified heterozygous marker alleles corresponding to the genes *Pi54* and *Pi1* and 3 carried only *Pi54*. All three were backcrossed to RP to yield 234 BC_2_F_1_ seeds. Two hundred individuals were subjected to FS, of which ninety-seven BC_2_F_1_ plants carried *Pi54*. These plants were analyzed at recombinant markers RM26746 (16.8), R11M23 (19.2), and RM144 (28.2), which helped select the best 20 BC_2_F_1_ lines carrying RP alleles at such loci. The progeny of selected BC_2_F_1_s from parallel backcrosses were also validated in the corresponding F_2_ generation using foreground markers (Figure 2).

#### Marker-assisted background selection

A polymorphism survey was carried out between RP *MB* and the three-gene donor line DHMAS 70Q 164-1b using 278 genome-wide SSR/STS markers, of which 96 markers were found to be polymorphic between the parents. Only 52 markers were assayed on the set of selected BC_2_F_1_ lines. Furthermore, 213 SNP markers were assayed, of which 71 polymorphic SNPs were considered for analysis of RPG recovery. Therefore, based on 123 markers, including 10 markers on carrier chromosome 11, the backcross-derived lines exhibited an RPG range between 55.65% and 93.55%. Fourteen BC_2_F_1_ lines were compared for overall genome-wide recovery, among which MB-DH-2, MB-DH-3, and MB-DH-12 recorded RPG recovery of 93.54%, 92.74%, and 92.74%. These lines carried RP alleles for the vital agronomic and quality-related loci such as grain number (*Gn1*), heading date (*Ghd7* and *Ghd8*), fragrance (*BADH2*), and waxiness (*Wx*) (Supplementary Tables S3 and S4).

### Development of pyramided lines

Inter-cross F_1_ (SKUA-528) was developed from pair mating of the NILs MB-IR-15 and MB-DH-2, carrying the genes *Pi9* and *Pi54*, respectively. F_1_ was self-pollinated to raise a population of 533 inter-cross F_2_ plants. The sub-population of 93 plants was retained based on rigorous phenotypic selection for earliness, short grain type, and aroma after cooking. The plants were raised in an inter-cross F_3_ generation and were evaluated for agronomic traits. Out of the 93 selections, marker-assisted foreground selection helped us identify 54 lines carrying the two genes *Pi9+Pi54*, 23 with *Pi9*, and 16 with *Pi54* under hetero- or homozygous conditions. Of these, eight, four, and nine plants were found homozygous for *Pi9+Pi54* (Supplementary Table S5), *Pi9* (Supplementary Table S6), and *Pi54* (Supplementary Table S7)*,* respectively.

**FIGURE 3:**
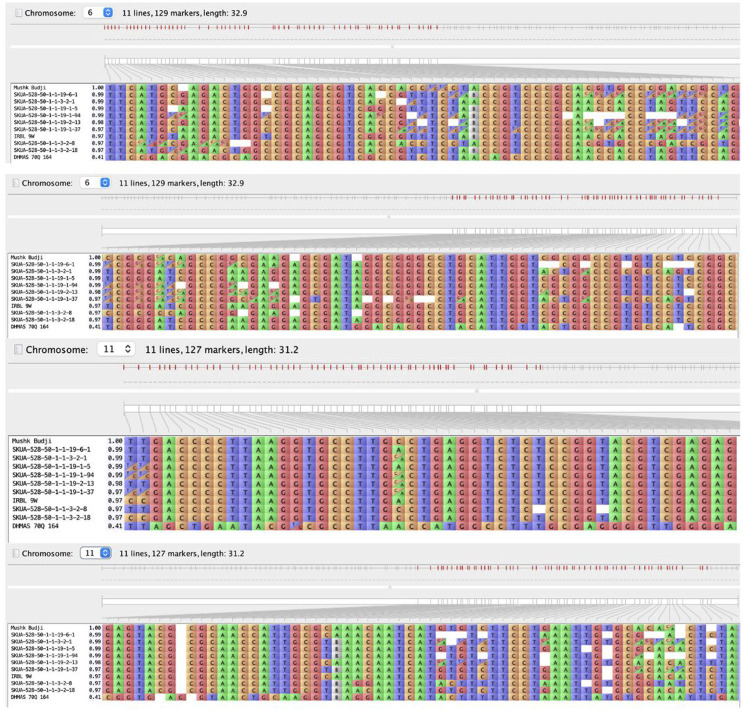
Graphical genotype of carrier chromosomes 6 and 11 depicting genome similarity of pyramided lines and NILs toward the recurrent parent, *Mushk Budji*. [The color codes differentiate individual bases (ATGC); homo- and heterozygotes designated as per the format generated through Flapjack (v1.21.02.04)].

Background analysis of selected homozygous lines carrying two- and single genes was carried out using 1,502 genome-wide KASP markers. The two-gene PLs, SKUA-528-50-1-1-3-2-18, SKUA-528-50-1-1-19-1-5, SKUA-528-50-1-1-19-1-37, and SKUA-528-50-1-1-19-1-94, carrying *Pi9+Pi54*, exhibited genome similarities of 84.59%, 90.10%, 88.26%, and 84.00%, respectively, with the recurrent parent *MB*. SKUA-528-50-1-1-19-6-1 and SKUA-528-50-1-1-19-2-13 both carried *Pi9* with the recurrent parent genome similarity of 89.25 and 90.95, respectively. The lines harboring *Pi54* included SKUA-528-50-1-1-3-2-1 and SKUA-528-50-1-1-3-2-8 with the genome similarity of 90.10 and 86.49, respectively. The lines carrying *Pi9* singly or in combination carried the resistance-specific allele within 3.07 Mb between the flanking markers gs_id994_ff (7.02 Mb) and gs_id1007_fn (10.09 Mb) (Figure 3). However, the line SKUA-528-50-1-1-19-1-5 (*Pi9+Pi54*) had an extra 0.51 Mb donor segment at the target locus. Likewise, the lines bearing the resistance-specific allele of *Pi54* were characterized with a small linkage drag of 0.9 Mb between the markers gs_id1779_ff (20.43 Mb) and gs_id1784_fn (21.32), except for SKUA-528-50-1-1-3-2-8 (*Pi54*), which carried a 6 Mb genomic region around the target *Pi54* (Figure 3). Moreover, the overall recovery on chromosome 6 was lower (72.07%) than the average genome recovery (87.97%) based on all 12 chromosomes across all eight lines (Supplementary Table S8).

**FIGURE 4:**
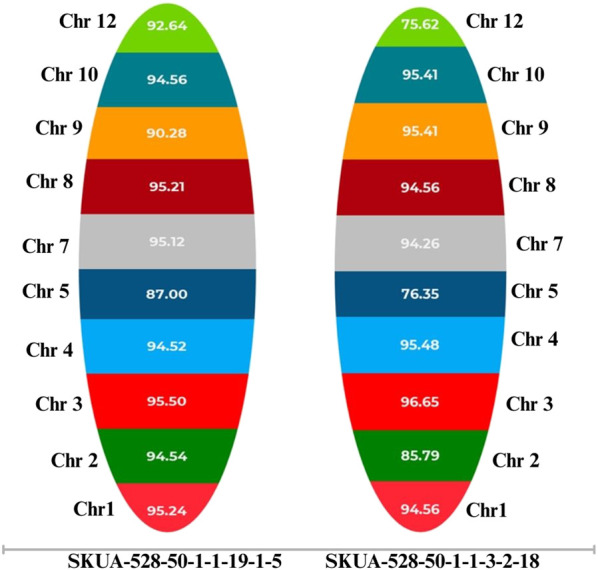
The colors represent non-carrier chromosomes; the bar width is in proportion to the percent RPG recovery (%).

Whole genome sequencing was carried out for the lines selected on the basis of genome recovery estimated using KASP markers. The estimation of genomic recovery was carried out based on 15,463 SNPs with MAF (minor allelic frequency) >40%, covering a physical map length of 369.43 Mbp. The genomic distribution and similarity map are provided in Supplementary Figures S1, S2. Among the lines carrying genes, *Pi9+Pi54*, SKUA-528-50-1-1-19-1-5 recorded the highest recovery of 93.46%, followed closely by SKUA-528-50-1-1-3-2-18 (90.41%), SKUA-528-50-1-1-19-1-94 (89.92%) and SKUA-528-50-1-1-19-1-37 (89.24%). Chromosome-wise analysis highlighted that while chromosomes 1, 3, 4, and 7 generally displayed high recovery across backcross lines; chromosomes 5, 9, 10, and 12 comparatively showed reduced recovery (Figure 4; Supplementary Tables S9 and S10).

**FIGURE 5:**
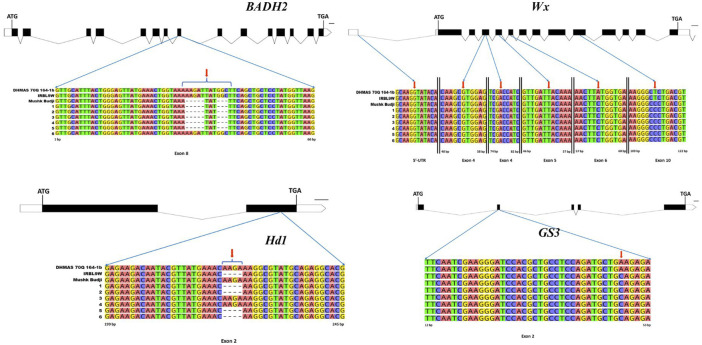
Sequence level validation of pyramided lines for important genes related to early flowering and cooking quality. [DHMAS 70Q 164-1b; IRBL9W; Mushk Budji; 1, SKUA-528-50-1-1-19-1-94; 2, SKUA-528-50-1-1-19-2-13; 3, SKUA-528-50-1-1-19-1-5; 4, SKUA-528-50-1-1-3-2-18; 5, SKUA-528-50-1-1-19-1-37; 6, SKUA-528-50-1-1-3-2-8].

In addition to the general assay for the estimation of RPG recovery using genome-wide markers, a set of key genes associated with quality and adaptability traits were characterized in PLs and the three parents. The temperate rice landrace MB, well-known for its unique aroma and eating quality, interestingly carried the *Wx* ^*in*^ allele (typically found in tropical *japonica*). The allele was successfully retained in all the PLs in contrast to donor lines IRBL9W and DHMAS 70Q 164-1b carrying *Wx* ^*a*^. For the *BADH2* underlying aroma, MB and PLs possessed the characteristic 8-bp deletion responsible for fragrance, whereas both the donor lines carried the functional (non-aromatic) *BADH2* allele, with the exception of line SKUA-528-50-1-1-19-2-13. In the case of the *Rc* gene controlling pericarp color, both DHMAS 70Q 164-1b and the recurrent parent carried the non-functional 14-bp deletion, while IRBL9W had the functional allele; with the exception of SKUA-528-50-1-1-19-2-13, which retained the red pericarp wild allele, other PLs carried the allele for white kernel. At the *GS3* locus determining grain size, both the donor lines carried the long-grain-specific allele, while MB and PLs carried the shorter-grain allele. For another locus, *LABA1*, which confers the awned phenotype, MB and IRBL9W carried the wild-type allele, while DHMAS 70Q 164-1b had a 1-bp deletion; most PLs retained the wild-type allele, except SKUA-528-50-1-1-3-2-8 and SKUA-528-50-1-1-19-2-13, which carried the deletion and exhibited the awnless phenotype. For the heading date (*Hd1),* MB and DHMAS 70Q 164-1b carried the functional allele, whereas IRBL9W carried a 4-bp deletion that was transmitted to four PLs. The *Hd4* (*Ghd7*) functional allele was found across all lines, while *Hd5* showed a 1-bp deletion in both donor parents and the NIL, SKUA-528-50-1-1-3-2-8. The gene interactions suggested that PLs carrying the 4-bp *Hd1* deletion in the background of functional *Hd4* and *Hd5* alleles are likely to flower earlier than the recurrent parent in Kashmir’s long-day ecology. Cold tolerance genes further differentiated the lines as at *COLD1*, MB and PLs carried the chilling-tolerant A allele, whereas two donors, PL SKUA-528-50-1-1-3-2-18 and individual *Pi54* carrying line, SKUA-528-50-1-1-3-2-8, carried the T allele. At *COLD6*, DHMAS 70Q 164-1b, MB, and the derived lines (except SKUA-528-50-1-1-3-2-8) had seven CTC repeats conferring higher cold tolerance, while IRBL9W carried (CTC)_6_ (Figure 5; Supplementary Figure S3).

### Evaluation of PLs for agronomic performance

**FIGURE 6:**
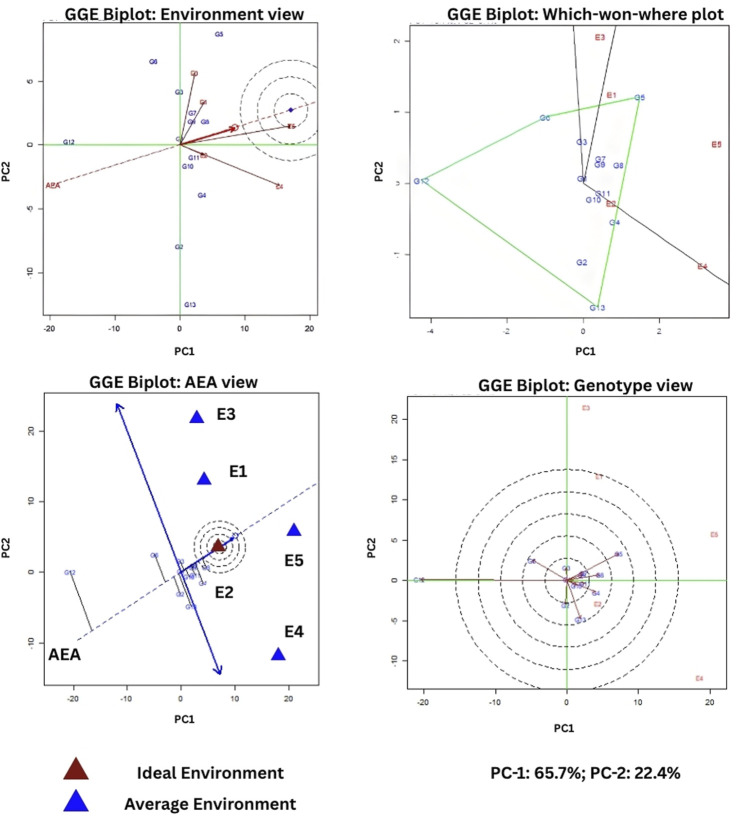
GGE biplot analysis of selected backcross derived lines across five environments. [Genotype designation: G5, SKUA-528-50-1-1-3-2-1; G8, SKUA-528-50-1-1-19-2-13; G7, SKUA-528-50-1-1-3-2-14; G3, SKUA-528-50-1-1-3-2-18; G9, SKUA-528-50-1-1-19-5-3; G11, Mushk Budji; G1, SKUA-528-50-1-1-19-1-5; G4, SKUA-528-50-1-1-19-6-1; G10, SKUA-528-50-1-1-19-1-37; G13, IRBL9W; G2, SKUA-528-50-1-1-19-1-94; G6, SKUA-528-50-1-1-3-2-8; G12, DHMAS 70Q 164-1b].

GGE biplot analysis of 10 NILs (intercross BC_2_F_4:6_), along with the three parents carried across a set of environmental locations, reflected significant differences in mean yield performance. The environment view revealed that arrow projections E4 and E5 facilitated a sharp discrimination across the NILs in contrast to the three other environments in terms of grain yield. E3 was found to be significantly distinct compared to E4 and E5, as confirmed from the wide angle of the environmental vector. Overall E1, E2, E4, and E5 represented a distinct mega-environment. The ‘which-won-where’ biplot analysis revealed that genotypes SKUA-528-50-1-1-19-1-5, SKUA-528-50-1-1-3-2-8, SKUA-528-50-1-1-3-2-14, SKUA-528-50-1-1-19-2-13, SKUA-528-50-1-1-19-5-3, and recurrent parent MB performed better in the traditional high-altitude environments E4 and E5. The donor parents DHMAS 70Q 164 did not perform well in any of the locations. Meanwhile, NIL SKUA-528-50-1-1-3-2-18 and SKUA-528-50-1-1-3-2-1 performed better in E1 and E3, respectively, and the donor IRBL9W had more adaptation toward high altitudes. The average environment coordinate (AEC) or average environment axis (AEA) view of the GGE biplot helped rank the genotypes in descending order of their mean performances and stability across environments. The arrangement of the genotypes was as follows: SKUA-528-50-1-1-3-2-1 > SKUA-528-50-1-1-19-2-13 > SKUA-528-50-1-1-3-2-14 = SKUA-528-50-1-1-3-2-18 > SKUA-528-50-1-1-19-5-3 > MB > SKUA-528-50-1-1-19-1-5 > SKUA-528-50-1-1-19-6-1 > SKUA-528-50-1-1-19-1-37 > IRBL9W > SKUA-528-50-1-1-19-1-94 > SKUA-528-50-1-1-3-2-8 > DHMAS 70Q 164-1b. The AEA view demonstrates that the genotypes closer to the arrow tip possessed higher mean yield and those nearer the line showed greater stability. The genotype view highlights the ideal genotype position: genotypes near this point (e.g., SKUA-528-50-1-1-3-2-1 SKUA-528-50-1-1-19-6-1) combine high performance and stability across test locations. Most of the NILs were plotted in high-altitude environments and reflected their maturity period *at par* with the MB parent. The donors were plotted away from the main sector on the plot because of their late maturity trait. Except for the SKUA-528-50-1-1-3-2-8, all other NILs showed earliness under target environments. AEC grouped the NILs and RP MB in one sector of the biplot and partitioned the donor parents separately on the opposite side of the perpendicular. Principal component analysis was carried out over a set of NILs for 12 agronomic, physico-chemical, and cooking quality traits. PC-1 and PC-2 together explained 88.1% of total variability, with PC-1 accounting for 65.7%. In a PCA biplot, most of the NILs are grouped in a single cluster and centered around the recurrent parent, whereas the two donor parents are plotted within two different quadrants (Figure 6). All the NILs recorded grain yield at par with the RP (18.78 g/plant), although five recorded the highest mean yield across locations. The field view of the pyramided line SKUA-528-50-1-1-19-1-5 (*Pi9+Pi54*) is documented in Supplementary Figure S4.

### Evaluation of PLs for physico-chemical and cooking quality traits

**FIGURE 7:**
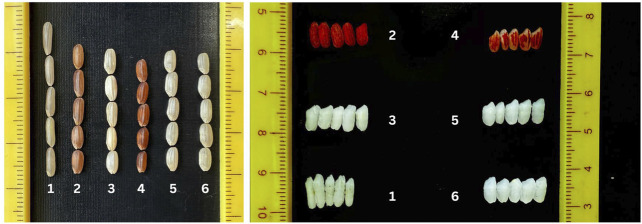
Kernel dimensions and cooking quality of pyramided lines. [1, DHMAS 70Q 164-1b (*Pi54*); 2, IRBL9W (*Pi9*); 3, Mushk Budji; 4, SKUA-528-50-1-1-19-2-13 (*Pi9*); 5, SKUA-528-50-1-1-19-1-5 (*Pi9+Pi54*); 6, SKUA-528-50-1-1-3-2-18 (*Pi9+Pi54*)].

The donor lines DHMAS 70Q 164 and IRBL9W were significantly different compared to the recipient parent MB and the PLs with respect to the traits KLBC, LBR, KER, amylose content, and aroma. The kernel shape, physico-chemical properties, and cooking quality of the derived lines were *at par* with MB. The donor for *Pi54* had intermediate amylose content, while the lines and the parent MB carried low amylose. The derived lines carried a similar aroma to the recurrent parent (Table 1; Figure 7).

**TABLE 1:** Mean performance of pyramided lines for physico-chemical and cooking quality traits across different locations.

S. No.	Line ID	Gene combination	KLBC (mm)	LBR	KLAC (mm)	KER	GC (mm)	AC (%)	ASV	Aroma	Kernel shape
1	SKUA-528-50-1-1-19-1-5	*Pi54+Pi9*	4.78 ± 0.05^c^	1.64 ± 0.03^c^	8.05 ± 0.04^bc^	1.63 ± 0.02^ab^	88.50 ± 1.79^c^	16.12 ± 0.26^e^	4	2	Short medium bold
2	SKUA-528-50-1-1-3-2-18	*Pi54+Pi9*	4.80 ± 0.05^c^	1.70 ± 0.02^c^	8.10 ± 0.05^c^	1.7 ± 0.03^ab^	87.75 ± 1.82^c^	16.8 ± 0.16^bc^	4	2	Short medium bold
3	SKUA-528-50-1-1-19-1-94	*Pi54+Pi9*	4.80 ± 0.03^c^	1.70 ± 0.04^c^	8.27 ± 0.06b	1.7 ± 0.01^a^	82.75 ± 1.71^c^	16.44 ± 0.10^cde^	4	2	Short medium bold
4	SKUA-528-50-1-1-19-1-37	*Pi54+Pi9*	4.74 ± 0.04^c^	1.63 ± 0.02^c^	7.93 ± 0.04^bc^	1.67 ± 0.01^ab^	92.75 ± 1.40^c^	16.92 ± 0.16^c^	4	2	Short medium bold
5	SKUA-528-50-1-1-3-2-1	*Pi54*	4.66 ± 0.03^c^	1.60 ± 0.02^c^	7.97 ± 0.04^bc^	1.7 ± 0.02^a^	88.25 ± 2.37^c^	16.2 ± 0.16^cd^	4	2	Short medium bold
6	SKUA-528-50-1-1-3-2-8	*Pi54*	4.82 ± 0.05^c^	1.70 ± 0.02^c^	8.23 ± 0.02^bc^	1.73 ± 0.02^a^	90.75 ± 1.69^c^	16.02 ± 0.14^cde^	4	2	Short medium bold
7	SKUA-528-50-1-1-3-2-14	*Pi54*	5.02 ± 0.05^c^	1.67 ± 0.02^c^	8.10 ± 0.06^bc^	1.63 ± 0.02^a^	86.25 ± 2.12^c^	17.04 ± 0.18^cd^	4	2	Short medium bold
8	SKUA-528-50-1-1-19-2-13	*Pi9*	4.94 ± 0.04^c^	1.57 ± 0.03^c^	8.12 ± 0.03^bc^	1.67 ± 0.02^a^	84.50 ± 1.01^c^	15.94 ± 0.15^de^	4	2	Short medium bold
9	SKUA-528-50-1-1-19-5-3	*Pi9*	4.94 ± 0.06^c^	1.70 ± 0.03^c^	8.28 ± 0.02^bc^	1.73 ± 0.02^a^	88.25 ± 2.03^c^	16.08 ± 0.11^cde^	4	2	Short medium bold
10	SKUA-528-50-1-1-19-6-1	*Pi9*	4.74 ± 0.05^c^	1.67 ± 0.04^c^	8.10 ± 0.04^bc^	1.7 ± 0.01^ab^	88.75 ± 1.90^c^	16.38 ± 0.17^cde^	4	2	Short medium bold
11	Mushk Budji	*-*	4.97 ± 0.03^c^	1.80 ± 0.01^c^	8.24 ± 0.02^bc^	1.7 ± 0.01^a^	89.00 ± 1.19^c^	16.14 ± 0.12^cde^	4	2	Short medium bold
12	DHMAS 70Q 164-1b	*Pi54*	7.23 ± 0.02^a^	3.00 ± 0.02^a^	10.60 ± 0.05^a^	1.43 ± 0.01^bc^	55.00 ± 0.60^c^	20.8 ± 0.09^a^	​	5	Medium slender
13	IRBL9W	*Pi9*	6.06 ± 0.05^b^	2.26 ± 0.05^b^	7.92 ± 0.05^c^	1.23 ± 0.02^c^	69.25 ± 0.76^b^	18.54 ± 0.09^b^	4	0	Medium bold

KLBC, kernel length before cooking; LBR, length to breadth ratio; KLAC, kernel length after cooking; KER, kernel elongation ratio; GC, gel consistency; AC, amylose content; ASV, alkali spreading value. The superscript letters on means ± S.E. within a column indicate significant differences at *p* ≤ 0.05 (under *post hoc* multiple comparison tests). Means sharing the same letter (e.g., both with ‘a’) are not significantly different from each other; means with different letters (e.g., ‘a’ vs. ‘b’) are significantly different (*p* ≤ 0.05); means with double digits (e.g., ‘bc’ connotes similarity to both ‘b’ and ‘c’).

### Disease reaction of PLs

**FIGURE 8:**
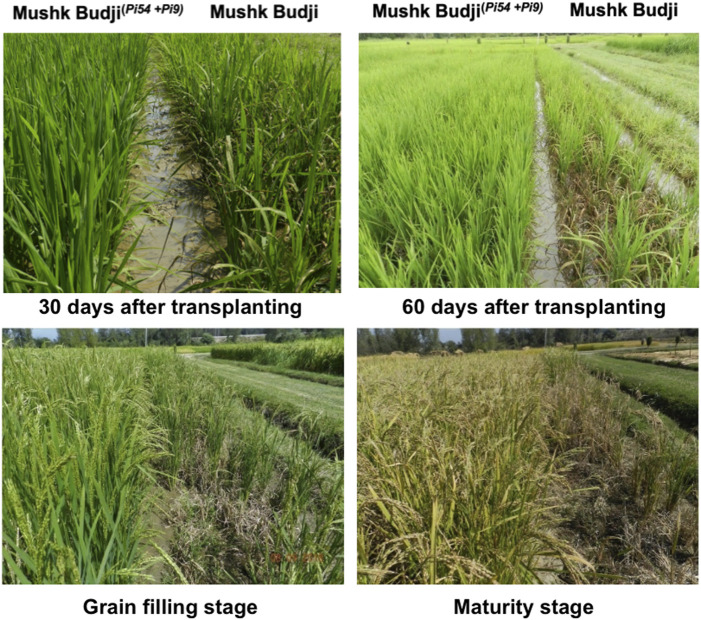
Resistance response of the gene pyramided line SKUA-528-50-1-1-19-1-5 under rice blast hot spot conditions at MRCFC, SKUAST-K, Khudwani campus.

The four PLs (*Pi9+Pi54*) and two each NILs for genes *Pi9* and *Pi54* were screened for blast disease reaction using two diagnostic isolates, Mo-nwi-kash-32 and SKUA-Mo-3 of *M. oryzae*, under controlled conditions with the corresponding two donors and RP as checks. The PLs SKUA-528-50-1-1-19-1-5 and SKUA-528-50-1-1-19-1-94 carrying *Pi9+Pi54* expressed an immune response to both the isolates, whereas SKUA-528-50-1-1-3-2-18 showed a hypersensitive reaction under controlled conditions. The ‘hypersensitivity’ has been described as a feature that may manifest itself as a ‘superficial macroscopic appearance of cell death’ (Balint-Kurti, 2019). The NILs carrying *Pi9* were resistant against the two isolates with disease scores of 0 and 1. However, the *Pi54* NILs SKUA-528-50-1-1-3-2-1 and SKUA-528-50-1-1-3-2-8 recorded a disease score of 3 and 2, respectively, against the isolate, SKUA-Mo-3. A set of lines was also tested at the uniform Blast Nursery at the hot spot location of Khudwani. All lines exhibited a resistance response to the prevalent isolates, whereas the RP *MB*, planted as a check, showed heavy disease severity with a score of 7–9 (Table 2; Figure 8).

**TABLE 2:** Disease reaction of pyramided lines against *Magnaporthe oryzae* isolates under controlled and field conditions.

S. No.	Plant ID	Gene combination	Reaction against *M. oryzae* isolates under controlled conditions^a^		Reaction under field hotspot conditions^b^
​	​	​	Mo-nwi-kash-32	SKUA-Mo-3	​
1	SKUA-528-50-1-1-19-1-5	*Pi9+Pi54*	0	0	0
2	SKUA-528-50-1-1-3-2-18	*Pi9+Pi54*	1	1	0
3	SKUA-528-50-1-1-19-1-94	*Pi9+Pi54*	0	0	0
4	SKUA-528-50-1-1-19-1-37	*Pi9+Pi54*	0	1	0
5	SKUA-528-50-1-1- 3-2-1	*Pi54*	0	3	2
6	SKUA-528-50-1-1-3-2-8	*Pi54*	0	2	2
7	SKUA-528-50-1-1-19-2-13	*Pi9*	0	1	0
8	SKUA-528-50-1-1-19-6-1	*Pi9*	0	1	0
9	IRBL 9W	*Pi9*	0	0	0
10	DHMAS 70Q 164-1b	*Pi54+Pi1+Pita*	0	0	0
11	*Mushk Budji*	-	5	5	7-9

^a^ Leaf blast scoring was performed as the per 0–5 scale provided by Mackill and Bonman (1992); Score 0–2: R; 3–5: S.

^b^ Leaf blast scoring was performed as the per 0-9 scale of International Rice Research Institute (2013).

## Discussion

*Mushk Budji* is a short-grained aromatic rice grown in mid-altitude regions (1,700–1,900 msl) of the Kashmir Valley. The landrace is known for its lustrous texture, taste, and pleasant aroma (Khan et al., 2017). MB has a huge demand, sells at a premium price, and is used widely during festive occasions. However, unfortunately, the cultivar suffered a declining trend in area during the last 2 decades owing to its high susceptibility to rice blast (Najeeb et al., 2018). The pathogen *Magnaporthe oryzae* can cause heavy yield losses, ranging from 70%–100%, under disease-favoring conditions. It requires repeated fungicidal applications to prevent the spread of the disease, which is not only uneconomic but also ecologically unsafe. The traces of fungicide are retained in the rice kernel, which can be of hazardous proportions and, therefore, unhealthy. Keeping this in view, the two major dominant genes *Pi9* and *Pi54* were pyramided into the genetic background of MB. Among several known blast resistance genes, *Pi9* and *Pi54* have been known to be among the most effective resistance genes under Kashmir conditions (Khan et al., 2018; Amin et al., 2025). The genes *Pi9* and *Pi54* originating from two separate donors, viz., IRBL9W and DHMAS70Q 164-1b, respectively, were transferred following the MABB approach. MABB has proven to be an effective strategy with an added advantage over the conventional backcrossing, especially for pyramiding two or more genes (Ellur et al., 2016; Yang et al., 2019; Latif et al., 2025). The effect of individual genes in a single host cannot be easily judged phenotypically in the presence of another gene with a similar effect, particularly when the pathogen used for phenotypic assays exhibits overlapping specificities for the constituent genes, making the pyramiding of genes within a common genetic background practically untenable. Therefore, the use of precisely placed markers linked to the gene of interest is expected to help in tracing the presence of constituent alleles in a gene pyramid. Apart from stacking the genes together, the approach helps validate the comparative performance of the gene combination against the pathogen race.

Marker-assisted foreground selection for the genes *Pi9* and *Pi54* was carried out using the gene-based markers Pro-9 and Pi54 MAS*,* respectively (Qu et al., 2006; Ramkumar et al., 2011). The use of gene-based markers allows the transfer of genes of interest with high precision and accuracy. Theoretical expectations in using a linked marker (in the heterozygous phase) for the foreground section are such that within an interval of 10 cm distance between flanking markers and the gene of interest, there lies a 0.024 probability of losing the gene after a single generation, which goes up to 0.1140 after five generations. The above expectation is supported by Anilkumar et al. (2023), where it is advocated that genic markers may increase the prediction accuracy of genomic selection for quantitative traits. In BC_2_F_1_-derived inter-cross F_3_, a total of 93 plants were put to foreground selection, of which 8 were found homozygous for *Pi9+Pi54*, 4 for *Pi9*, and 9 for *Pi54*, respectively. The marker Pi54 MAS amplified a 216 bp fragment specific to *Pi54* resistance and a 359 bp allele for susceptible plants. There happens to be a deletion of 144 bp in the exonic region of a gene in resistant lines. The marker Pro-9 amplified a 128 bp fragment specific to *Pi9* resistance and a 150 bp allele for susceptible plants. The target control rate (Hospital, 2009) is expected to be maximum for gene-based markers so that the trait under selection is precisely transferred in all the backcross progenies, which amplify the desirable allele.

In addition to the markers for foreground selection, polymorphic SSR markers and 1,501 genome-wide KASP markers were utilized for marker-assisted background selection ((Hospital et al., 1997) to achieve rapid recovery of the recurrent parent genome. The RPG recovery ranged from 84.00% for SKUA528-50-1-1-19-1-94 (*Pi9+Pi54*) to the maximum value of 90.95% for SKUA528-50-1-1-19-2-13 (*Pi9*). While carrying out background selection, no preferential selection of individuals was made based on RPG recovery on carrier chromosomes as this would likely have resulted in lower overall RPG content. As reported by Newsholme et al. (2000), the stringent selection on carrier chromosomes may reduce selection pressure on non-carrier chromosomes, which constitute a major part of the genome. In this regard, the selection pressure on carrier chromosomes was moderated by allowing scoring for non-carrier chromosomes in equal measure. To control the linkage drag, the number of markers used on carrier chromosome was kept higher than the genomic average. Overall gain in the proportion RPG depends upon several factors and includes the generation in which selection is exercised (early or late) and the number of markers. To balance the better consequences out of these considerations we performed selections in BC_2_F_1_ and then again in the inter-cross populations. This was possible after we practiced early selection based on easily observable (phenotypic) traits. The practice of employing phenotypic selection along with selection for background markers has been previously adopted in several studies (Rajpurohit et al., 2011; Singh et al., 2012; Khanna et al., 2015). This provides the chance of eliminating undesirable drag to some extent, whether linked or unlinked to the target gene. This was followed by selection based on markers linked to vital traits, after which genome-wide MAS was extended by adopting the ‘two-stage selection’ process described by Frisch et al. (1999). According to their simulation method, the 10th percentile (Q10) of the distribution of the recurrent parent genome (RPG) averages approximately 86.1% in BC_2_ for ∼20 genotypes screened at 440 MDPs (marker data points) and an introgression of a single allele. In our experiment, we produced 1,320 MDPs and achieved an average RPG of 86.81%, as per the expectation for the transfer of *Pi9*. Similarly, for *Pi54* carrying NILs, selection was carried out using 1736 MDPs with an RPG of 88.47%. It shows that the two-stage selection process in the development of NILs has been efficient enough to yield high genome recovery in NILs, which were inter-crossed to obtain PLs. The markers Gn1a-3_SNP_nn_2 and Gn1a_1_SNP_nn_1 linked to grain number locus Gn1 (Ashikari et al., 2005) at 5.1 Mb of chromosome 1 were polymorphic between donor DHMAS 70Q 164-1b and MB. All the selected NILs in BC_2_F_1_ showed the RP allele (T) at the grain number locus with G and C SNPs at two loci, respectively. The trait GP recorded a clear contrast between RP/NILs and the *Pi54* donor. The KASP marker, FGR8_SNP3, linked to fragrance, amplified T in 13 out of 20 NILs carrying *Pi54* against non-aromatic donor with the C allele. A marker GS5_03_1_SNP_nn_1 on chromosome 5 linked to grain dimension (Li et al., 2011) showed G/A polymorphism and had RP allele at all but one *Pi54* NIL. The SNPs (Waxy_SNP, Amy_W2_R_1, and Amy_RM190_func_1) linked to the *Wx* locus (He et al., 1999; Wang et al., 2007) at 1.6 Mb on chromosome 6 amplified the TAC haplotype for four and the CGA haplotype for eight out of the 20 *Pi54* NILs. All NILs carried the RP-specific allele for KASP marker Ghd7_05_SNP_ff_1 at the *Ghd-7* locus. The *Ghd-7* locus controls heading date at 9.3 Mb on chromosome 7 (Xue et al., 2008). Natural mutants with reduced *Ghd* function enable rice to be cultivated in temperate regions (Zhang et al., 2021). This was a remarkable trait where recovery was essential because the male parent DHMAS 70Q 164-1b flowered 25–30 days later than RP MB. The *Pi9* NILs were also selected based on RM190 marker linked to the *Wx* allele on carrier chromosome 6 and helped us obtain low amylose lines.

*Mushk Budji* is known for its pleasant aroma, taste, and distinct short and bold kernels. However, DHMAS70Q 164-1b, the donor for the blast resistance gene *Pi54* used in the present study, is a doubled haploidy-derived semi-fine grained rice genotype derived from the rice cultivar *Tetep*. The genotype showed a wide difference of at least 2.43 mm in milled rice length (KLBC) and 2 mm in cooked kernel length (KLAC) compared to MB. The two parents, MB and DHMAS 70Q 164-1b, belong to *temperate japonica* and *indica* ecotypes, respectively (Shikari et al., 2021). Furthermore, a donor for *Pi9,* IRBL9W is *japonica*-type monogenic line with red pericarp. Therefore, as backcross-derived lines were expected to segregate for grain shape, cooking quality, aroma, and several other traits, the recovery of features specific to recurrent parent MB was a primary target. The traits, such as aroma, kernel length after cooking (KLAC), and kernel elongation ratio (KER), follow complex inheritance patterns and are supposed to be governed by major and several minor-effect QTLs (Amarawathi et al., 2008). The stringent phenotypic selection for these traits was employed in conjunction with marker-assisted background selection in accordance with the works of Singh et al. (2012). The kernel shape, physico-chemical properties, and cooking quality of the derived lines were at par with *MB*. The donor for *Pi54* had intermediate amylose content, while the lines and the parent *MB* carried low amylose. The performance of PLs and NILs for most of the agro-morphological traits was, in general, similar to that of the recipient parent, MB. However, five lines produced significantly higher mean yield across locations. The data showed that there was no penalty for yield or grain quality in selected PLs. The fragrance trait in MB can be explained by a recessive gene *badh2* located on chromosome 8, which encodes a compound, 2-acetyl 1-pyrroline (Cruz and Khush, 2000). The marker profile of the *badh2* and *Wx* allele showed a correlation with the phenotypic expression of the PLs for aroma and amylose content.

GGE biplot analysis was carried out to investigate the agronomic performance of derived lines within and outside the target regions. This is a robust technique for depicting genotypes in a which-won-where fashion as it combines genotype (G) main effects and genotype x environment (GE) components to evaluate the genotype performance and designate the mega-environments supporting such genotypes (Yan et al., 2000). The five genotypes, which included a single, two-gene pyramid (*Pi9+Pi54*), two lines each carrying *Pi9* and *Pi54* and MB, performed better in the traditional high-altitude environments (E4 and E5).

The final set of selected PLs and NILs was screened under artificial conditions and showed resistance against the two diagnostic isolates of *M. oryzae*, viz., Mo-nwi-kash-32 and SKUA-Mo-3, used by us previously (Khan et al., 2018). Furthermore, a set of these selected lines was also tested under the Uniform Blast Nursery at the hot spot location of Khudwani. All lines exhibited a resistance response to the prevalent isolates, whereas the RP MB, planted as a check, showed heavy disease severity with a score of 9. This authenticates our choice of genes in constituting PLs/NILs.

The whole genome re-sequencing helped us perform allelic characterization of quality and adaptability-related genes in NILs. The sequence information on the derived NILs and the parents helped us confirm the underlying genetics of important quality and adaptability traits in *Mushk Budji*. Furthermore, it helped us validate the successful recovery of desirable alleles for aroma, resistant starch, cold tolerance, and early flowering genes in blast gene PLs with a similar genetic constitution as that of MB. For instance, all the PLs successfully retained the *Wx* ^*in*^ allele of MB in contrast to the donor lines IRBL9W and DHMAS 70Q 164-1b, both of which carried *Wx* ^*a*^. However, the alkali locus, known to the control gelatinization temperature trait, the donor, the recurrent parent, and all the PLs carried the *ALK* ^*c*^ allele. The *ALK* ^*c*^ allele has been reported to be associated with increased levels of resistant starch (RS) (Parween et al., 2020), which carries a direct relation to low glycemic index in rice (Zaffer et al., 2025). The aroma in rice is primarily governed by the *BADH2* gene. An eight-base pair deletion in exon 7 of this gene has been shown to cause a premature truncation of betaine aldehyde dehydrogenase enzyme, which leads to the accumulation of 2-acetyl-1-pyrroline (2AP), a key aromatic compound responsible for the characteristic fragrance of rice (Sakthivel et al., 2009). In addition to this deletion, mutations at several other positions within the *BADH2* gene have also been reported to confer aroma in rice (Kovach et al., 2009). Presently, both the donor lines carried the functional *BADH2* enzyme, whereas MB possessed the characteristic 8-bp deletion associated with aroma, except SKUA-528-50-1-1-19-2-13. *Rc*, a domestication-related gene encoding a basic helix-loop-helix (bHLH) transcription factor, controls red pericarp color in rice. A 14-bp deletion in exon 6 disrupts the bHLH domain, resulting in a loss of function (Sweeney et al., 2006). Since one of the parents (IRBL9W for *Pi9*) carried the wild-type red pericarp (*Rc*) phenotype, the PLs, except one line, carried characteristic white kernel allele similar to that of MB (Figure 7). GS3 is a major QTL in rice that plays a crucial role in determining grain size and weight (Fan et al., 2006). Here, both the donor lines carried the long-grain-specific allele (A) against RP MB, and all PLs carried the alternate allele (C). A critical mutation in exon 2 of the *GS3* gene is responsible for introducing a premature stop codon into the coding sequence, resulting in the synthesis of a truncated and non-functional *GS3* protein, thereby disrupting its role as a negative regulator of grain elongation, a cause of the long-grained phenotype. Another locus *LABA1*, which encodes a cytokinin-activating enzyme, confers long and barbed awns, and a frameshift deletion results in short and barbless awns (Hua et al., 2015), a trait that might be useful for improved milling processability. MB and donor IRBL9W were found to carry the wild allele for *LABA1*, while *Pi54* donor DHMAS 70Q 164-1b had a 1 bp deletion. The awned trait was retained in the selected PLs. Moreover, genes linked to adaptive traits such as heading date and cold tolerance were considered. For the heading date, three key genes (*Hd1*, *Hd4*, and *Hd5*) known to regulate flowering showed significant polymorphism between donors and RP. Beyond their primary function in regulating heading date, these genes exhibit pleiotropic effects on grain yield, plant height, and ecogeographical adaptation of rice (Zhang et al., 2015; Zong et al., 2021), so these genes are important for breeding programs aimed at improving performance across diverse environments. For *Hd1*, it was observed that both MB and DHMAS 70Q 164-1b possessed the functional allele, whereas IRBL9W carried a 4 bp deletion. This deletion has been transmitted to four of the PLs. The 4 bp deletion occurs within the second exon of the gene, resulting in the disruption of the conserved CCT domain of the Hd1 protein (Zong et al., 2021). The functional *Hd4* (syn: *Ghd7*), defined by T/G substitution (Kim et al., 2018), was present in parental lines and PLs. Similarly, *Hd5* carried a 1 bp deletion in both the donor parents, however, MB and PLs (except SKUA-528-50-1-1-3-2-8) retained the allele related to earliness (Kim et al., 2018). The three loci discussed here interact at the protein level to form a functional complex, which regulates the Ehd1–Hd3a/RFT1 pathway, a key pathway governing flowering initiation and heading date in rice (Zong et al., 2021). *Hd1* functions as a promoter of flowering, while *Hd4* acts as a repressor, irrespective of the day length. Interestingly, the presence of functional alleles of either *Hd4* or *Hd5* can alter the primary role of *Hd1*, shifting its function from flowering promoter to suppressor under long-day conditions. Based on this, it can be inferred that the PLs carrying the 4 bp mutation in *Hd1* are likely to flower earlier than those with the wild-type allele under naturally long-day conditions in Kashmir ecology as most PLs possessed functional *Hd4* and *Hd5* alleles. The two loci for cold tolerance, *COLD1* and *COLD6*, were surveyed. In *COLD1*, nucleotide position 15 of exon-IV plays a key role in determining chilling tolerance (Ma et al., 2015) and involves in a single A-to-T change, resulting in a lysine-to-methionine change. The donor lines carried the recessive (susceptible) allele, whereas MB and the PLs were validated for the chilling tolerance A allele. Similarly, the *COLD6* gene, which forms part of the chilling sensor complex and triggers the accumulation of 2′, 3′-cAMP, contributes to enhanced chilling tolerance. The CTC codon repeats within *COLD6* exon have been identified as contributing toward environmental adaptation (Luo et al., 2024), particularly the (CTC)_7_ allele, which has shown to be linked to higher chilling tolerance compared to the (CTC)_6_ allele. Presently, the selected PLs successfully retained the favorable (CTC)_7_ that potentially confers chilling tolerance. With the successful validation of favorable alleles for yield, quality, and adaptability in addition to major gene resistance, it became clear that rigorous phenotypic selection in the backcross generation is a very important step in the MABB program.

## Conclusion

The marker-assisted foreground selection and genome-wide KASP/SSR marker-based background selection helped us successfully pyramid blast resistance genes *Pi9* and *Pi54* into Mushk Budji. Whole genome sequencing of the selected PLs confirmed the retention of favorable alleles at key genetic loci, namely, *BADH2*, *Wx*, *Rc*, *Ghd7/Hd1/Hd5*, and *COLD1/COLD6*. We, for the first time, report the allelic control of aroma, earliness, cold tolerance, and starch quality in the landrace, *Mushk Budji*. We advocate the use of rigorous phenotypic selection for important agronomic and quality traits in early backcross generations and (or) the selection criteria based on trait-linked genes for achieving high recovery of phenotype and adaptability. Multi-environment screening facilitated the selection of final lines with stable resistance to *M. oryzae* with no penalty on yield or quality traits. The improved PLs (SKUA-528-50-1-1-19-1-5 and SKUA-528-50-1-1-19-1-94) and NILs (SKUA-528-50-1-1-3-2-18, SKUA-528-50-1-1-3-2-1, and SKUA-528-50-1-1-3-2-8) in the genetic background of MB could be promoted for general cultivation across temperate rice-growing ecologies of the region.

## Data Availability

The datasets generated for this study can be found in the NCBI Sequence Read Archive (SRA), under BioProject accession number PRJNA1397917.
