# Supplementary material for: Genomics-informed elucidation of trait-phenotype relationships and MABB approaches deliver major gene blast resistance in the aromatic rice landrace Mushk Budji
Source: Front Genet. 2026 Jan 22;16:1699333. doi: 10.3389/fgene.2025.1699333 (PMC12872327; doi:10.3389/fgene.2025.1699333)
Supplement: Supplementary file 2 [file DataSheet2.docx]

**Table S1. RPG recovery (%) of BC_2_F_1_ plants carrying blast resistance gene *Pi9***

| MB-IR-1 | MB- IR-2 | MB- IR-3 | MB-IR-4 | MB-IR-5 | MB-IR-6 | MB-IR-7 | MB-IR-8 | MB-IR-9 | MB-IR-10 | MB-IR-11 | MB-IR-12 | MB-IR-13 | MB-IR-14 | MB-IR-15 |
| --- | --- | --- | --- | --- | --- | --- | --- | --- | --- | --- | --- | --- | --- | --- |
| 85.63 | 93.68 | 95.40 | 91.95 | 93.10 | 80.46 | 81.03 | 78.74 | 83.33 | 83.33 | 81.03 | 90.23 | 90.80 | 77.01 | 96.55 |

**Table S2. Allelic profiling of BC_2_F_1_ plants carrying blast resistance gene *Pi9* for estimation of RPG recovery (%)**

| Marker Name | Chr. No. | Mb Pos | MB | IRBL9W | MB-IR-1 | MB- IR-2 | MB- IR-3 | MB-IR-4 | MB-IR-5 | MB-IR-6 | MB-IR-7 | MB-IR-8 | MB-IR-9 | MB-IR-10 | MB-IR-11 | MB-IR-12 | MB-IR-13 | MB-IR-14 | MB-IR-15 |
| --- | --- | --- | --- | --- | --- | --- | --- | --- | --- | --- | --- | --- | --- | --- | --- | --- | --- | --- | --- |
| RM580 | 1 | 10.5 | A | B | A | A | A | A | A | A | H | H | H | A | A | A | H | A | A |
| RM129 | 1 | 21.4 | A | B | H | A | A | A | A | A | A | A | A | A | H | A | A | A | A |
| R1M30 | 1 | 25.4 | A | B | A | A | A | A | A | A | A | A | A | A | A | A | A | A | A |
| R1M37 | 1 | 30.5 | A | B | B | A | A | A | A | A | A | A | A | A | A | B | A | A | A |
| Pi-37_SNP_ff_1 | 1 | 34.2 | A | B | B | A | A | A | A | A | B | B | B | A | B | B | B | B | A |
| R1M47 | 1 | 37.4 | A | B | A | A | A | A | A | A | A | A | A | A | A | A | A | A | A |
| RM 71 | 2 | 9.7 | A | B | A | A | A | A | A | A | A | A | A | A | A | A | A | A | A |
| S02057B | 2 | 14.5 | A | B | A | A | A | A | A | H | A | H | H | A | A | A | A | A | A |
| RM262 | 2 | 20.8 | A | B | A | A | A | A | A | A | A | A | A | A | A | A | A | A | A |
| S02085 | 2 | 21.4 | A | B | A | A | A | A | A | A | A | B | A | A | A | A | A | A | A |
| S02085 | 2 | 21.5 | A | B | A | A | A | A | A | H | A | A | A | A | A | A | A | A | A |
| RM517_SNP_nn_5 | 3 | 6.3 | A | B | A | A | A | A | A | A | A | A | A | A | A | A | A | A | A |
| qUbi_SNP_nn_4 | 3 | 7.2 | A | B | A | A | A | A | A | A | A | A | A | A | A | A | A | A | A |
| S03048 | 3 | 12.1 | A | B | A | A | A | A | A | A | A | A | A | A | A | A | A | A | A |
| R3M30 | 3 | 20.2 | A | B | A | A | A | A | A | A | A | A | A | A | A | A | A | A | A |
| S03136 | 3 | 34.0 | A | B | A | A | A | A | B | H | A | A | A | H | A | A | A | A | A |
| RM537_SNP_ff_1 | 4 | 0.2 | A | B | A | B | B | B | B | B | A | A | A | A | B | A | A | A | B |
| R4M17 | 4 | 11.8 | A | B | A | A | A | A | A | A | A | A | A | A | A | A | A | A | A |
| S04060 | 4 | 15.1 | A | B | A | A | A | A | A | A | A | A | A | A | A | A | A | A | A |
| Xa2_candidate4 | 4 | 31.9 | A | B | A | B | B | A | B | B | B | A | B | B | A | A | A | A | A |
| S04129B | 4 | 32.4 | A | B | A | B | B | A | B | B | B | A | B | B | A | A | A | A | A |
| R5M13 | 5 | 5.8 | A | B | A | A | A | A | A | A | A | A | A | A | A | A | A | A | A |
| OsR498G0510120000_SNP_ff_3 | 5 | 8.9 | A | B | B | A | A | B | A | B | B | B | B | B | B | B | B | B | B |
| R5M20 | 5 | 13.4 | A | B | A | A | A | A | A | A | A | A | A | A | A | A | A | A | A |
| RM190 | 6 | 1.7 | A | B | A | A | A | H | A | A | A | A | A | B | H | A | A | A | A |
| RM204 | 6 | 3.1 | A | B | H | H | A | H | A | A | A | A | A | A | A | A | A | H | A |
| RM584 | 6 | 3.3 | A | B | H | H | A | A | A | A | A | A | A | A | A | A | A | H | A |
| RM314 | 6 | 4.8 | A | B | A | A | A | A | A | A | A | A | A | A | A | A | A | H | A |
| RM253 | 6 | 5.4 | A | B | A | A | A | A | A | A | A | A | A | H | H | A | A | H | A |
| RM276 | 6 | 6.2 | A | B | A | A | A | A | A | B | B | H | A | H | H | A | A | H | H |
| RM19629 | 6 | 6.3 | A | B | A | A | A | A | H | B | B | B | A | H | H | A | A | H | H |
| RM19721 | 6 | 8.1 | A | B | A | A | A | H | H | B | B | B | A | A | A | H | H | H | H |
| RM19771 | 6 | 9.0 | A | B | H | H | H | H | H | B | B | B | A | A | A | H | H | H | H |
| AP-5659-5 | 6 | 10.4 | A | B | B | B | B | B | B | B | B | B | B | B | B | B | B | B | B |
| RM19923 | 6 | 12.3 | A | B | H | H | H | H | H | B | B | B | B | B | B | H | H | B | A |
| RM19959 | 6 | 13.0 | A | B | H | H | H | H | H | B | B | B | B | B | B | H | H | B | A |
| RM19970 | 6 | 13.2 | A | B | H | H | H | H | H | B | B | B | B | B | B | H | H | B | A |
| RM19974 | 6 | 13.3 | A | B | H | H | H | H | H | B | B | B | B | B | B | H | H | B | A |
| RM19979 | 6 | 13.5 | A | B | H | H | H | H | H | B | B | B | B | B | B | H | H | B | A |
| RM19980 | 6 | 13.5 | A | B | H | H | H | H | H | B | B | B | B | B | B | H | H | B | A |
| RM19983 | 6 | 13.7 | A | B | H | H | H | H | H | B | B | B | B | B | B | H | H | B | A |
| RM19986 | 6 | 13.8 | A | B | H | H | H | H | H | B | B | B | B | B | B | H | H | B | A |
| RM19997 | 6 | 14.2 | A | B | H | H | H | H | H | B | B | B | B | B | B | H | H | B | A |
| RM20028 | 6 | 15.7 | A | B | H | A | A | H | A | B | B | H | A | B | B | A | A | B | A |
| RM20190 | 6 | 19.9 | A | B | A | A | A | H | A | A | A | A | A | A | A | A | A | B | A |
| RM20338 | 6 | 23.2 | A | B | A | A | A | H | A | A | A | A | A | A | A | A | A | B | A |
| R6M44 | 6 | 26.0 | A | B | A | A | A | H | A | A | A | A | A | A | A | A | A | B | A |
| RM-30 | 6 | 27.2 | A | B | A | A | A | H | A | A | A | A | A | A | A | A | A | B | B |
| OsR498G0713985600_SNP_ff_1 | 7 | 10.4 | A | B | A | A | A | A | A | A | A | A | A | A | H | A | A | A | A |
| R7M37 | 7 | 23.7 | A | B | A | A | A | A | A | A | A | H | H | H | A | A | A | A | A |
| R7M7 | 7 | 63.5 | A | B | A | A | A | A | A | A | A | A | A | A | A | A | A | A | A |
| CRG4_SNP_nn_1 | 8 | 2.9 | A | B | A | A | A | A | A | A | B | A | A | A | A | A | A | A | A |
| Pi33_RM310_SNP2 | 8 | 5.0 | A | B | B | A | A | A | A | A | A | B | B | A | A | B | B | B | B |
| RM22804 | 8 | 11.3 | A | B | A | A | A | A | A | A | A | A | A | A | A | A | A | A | A |
| RM331_SNP_fn_1 | 8 | 12.3 | A | B | A | A | A | A | A | A | A | A | A | A | A | A | A | A | A |
| RM7102 | 8 | 13.2 | A | B | A | A | A | A | A | A | A | A | A | A | A | A | A | A | A |
| RM404 | 8 | 16.5 | A | B | A | A | A | A | A | A | A | A | A | A | A | A | A | A | A |
| RM23641 | 8 | 28.3 | A | B | A | A | A | A | A | A | A | A | A | A | A | A | A | A | A |
| RM72 | 9 | 3.2 | A | B | A | A | A | A | A | A | A | A | A | A | A | A | A | A | A |
| RM 72 | 9 | 3.2 | A | B | A | A | A | A | A | A | A | A | A | A | A | A | A | A | A |
| S09040B | 9 | 10.2 | A | B | A | A | A | A | A | A | A | A | A | A | A | A | A | A | A |
| S09073 | 9 | 18.4 | A | B | A | A | A | A | A | A | A | A | A | A | A | A | A | A | A |
| S10001 | 10 | 0.5 | A | B | A | A | A | A | A | A | A | A | A | A | A | A | A | A | A |
| S10003A | 10 | 0.8 | A | B | A | A | A | A | A | A | A | A | A | A | A | A | A | A | A |
| RM271 | 10 | 3.5 | A | B | A | A | A | A | A | A | H | H | H | A | A | A | A | A | A |
| RM216 | 10 | 4.6 | A | B | A | A | A | A | A | A | A | A | A | A | A | A | A | A | A |
| OsR498G1018206100_SNP_ff_1 | 10 | 4.7 | A | B | B | A | A | A | A | A | B | B | B | A | A | B | B | B | A |
| R10M17 | 10 | 9.0 | A | B | A | A | A | A | A | A | A | A | A | A | A | A | A | A | A |
| RM7102_SNP2 | 10 | 12.5 | A | B | B | A | A | A | A | B | A | B | A | B | B | B | B | B | A |
| R10M30 | 10 | 17.0 | A | B | B | A | A | A | A | H | A | B | A | B | B | B | B | B | B |
| R10M40 | 10 | 19.5 | A | B | H | A | A | A | A | A | A | A | A | A | A | A | A | A | A |
| HVSSR11-12 | 11 | 2.5 | A | B | A | A | A | A | A | A | A | A | A | A | A | A | A | A | A |
| RM202 | 11 | 7.5 | A | B | A | A | A | A | A | A | A | A | A | A | A | A | A | A | A |
| RM26746 | 11 | 16.8 | A | B | H | H | A | A | A | A | A | A | H | A | H | A | A | A | A |
| R11M23 | 11 | 19.2 | A | B | A | A | A | A | A | A | A | A | A | A | A | A | A | A | A |
| RM27000 | 11 | 21.8 | A | B | B | A | A | A | A | A | A | A | B | A | B | A | A | A | A |
| CKM -1 | 11 | 27.5 | A | B | A | A | A | A | A | A | A | A | A | A | A | A | A | A | A |
| RM144 | 11 | 28.2 | A | B | A | A | A | A | A | A | A | A | A | A | A | A | A | A | A |
| RM415 | 12 | 1.0 | A | B | A | A | A | A | A | A | A | A | A | A | A | A | A | A | A |
| RM453 | 12 | 2.6 | A | B | A | A | A | A | A | A | A | A | A | A | A | A | B | A | A |
| RM7619 | 12 | 4.8 | A | B | A | A | A | A | A | A | A | B | B | A | A | A | A | A | A |
| RM44 | 12 | 8.9 | A | B | A | A | A | A | A | A | A | A | A | A | A | A | A | A | A |
| RM27933 | 12 | 10.4 | A | B | A | A | A | A | A | A | H | H | A | A | A | A | A | A | A |
| PBA14 | 12 | 10.4 | A | B | A | A | A | A | A | A | A | A | A | A | A | A | A | A | A |
| OSM89 | 12 | 11.3 | A | B | H | A | A | A | A | A | A | A | A | A | A | A | A | A | A |
| RM27966 | 12 | 12.2 | A | B | A | A | A | A | A | A | A | A | A | A | A | A | A | A | A |
| RM27462 | 12 | 14.1 | A | B | A | A | A | A | A | A | A | A | A | A | B | A | A | A | A |
| RM19 | 12 | 14.3 | A | B | A | A | A | A | A | A | A | A | A | A | A | A | A | A | A |

**Table S3. RPG recovery (%) of BC_2_F_1_ plants carrying blast resistance gene *Pi54***

| MB-DH-1 | MB-DH-2 | MB-DH-3 | MB-DH-5 | MB-DH-6 | MB-DH-9 | MB-DH-10 | MB-DH-12 | MB-DH-14 | MB-DH-15 | MB-DH-16 | MB-DH-17 | MB-DH-19 | MB-DH-20 |
| --- | --- | --- | --- | --- | --- | --- | --- | --- | --- | --- | --- | --- | --- |
| 92.34 | 93.55 | 92.74 | 88.31 | 92.34 | 89.11 | 89.11 | 92.74 | 89.11 | 92.74 | 89.52 | 55.65 | 88.71 | 92.74 |

**Table S4. Allelic profiling of BC_2_F_1_ plants carrying blast resistance gene *Pi54* for estimation of RPG recovery (%)**

| Marker Name | Chr. No. | Mb Pos | Mushk Budji | DHMAS 70Q 164-1b | MB-DH-1 | MB-DH-2 | MB-DH-3 | MB-DH-5 | MB-DH-6 | MB-DH-9 | MB-DH-10 | MB-DH-12 | MB-DH-14 | MB-DH-15 | MB-DH-16 | MB-DH-17 | MB-DH-19 | MB-DH-20 |
| --- | --- | --- | --- | --- | --- | --- | --- | --- | --- | --- | --- | --- | --- | --- | --- | --- | --- | --- |
| Gn1a-3_SNP_nn_2 | 1 | 5.2 | A | B | A | A | A | A | A | A | A | A | A | A | A | A | A | A |
| Gn1a_1_SNP_nn_1 | 1 | 5.2 | A | B | A | A | A | A | A | A | A | A | A | A | A | A | A | A |
| RM580 | 1 | 10.5 | A | B | A | A | A | A | H | A | H | A | B | A | A | A | A | A |
| RM129 | 1 | 21.4 | A | B | A | A | A | A | A | A | A | A | A | A | A | A | A | A |
| R1M30 | 1 | 25.4 | A | B | A | A | A | A | A | A | A | A | A | A | A | A | A | A |
| R1M37 | 1 | 30.5 | A | B | A | A | A | B | A | A | A | A | A | A | B | B | B | A |
| Pi-37_SNP_ff_1 | 1 | 34.2 | A | B | A | A | A | B | A | A | A | A | A | A | B | B | B | A |
| R1M47 | 1 | 37.4 | A | B | A | A | A | B | A | A | A | A | A | A | B | B | B | A |
| RM14_SNP_ff_1 | 1 | 42.5 | A | B | A | A | A | A | A | A | A | A | A | A | A | A | A | A |
| Pit-2_SNP_nn_1 | 1 | 42.8 | A | B | A | A | A | A | A | A | A | A | A | A | A | A | A | A |
| Pit-2_SNP_nf_2 | 1 | 42.8 | A | B | A | A | A | A | A | A | A | A | A | A | A | A | A | A |
| RM 71 | 2 | 9.7 | A | B | A | A | A | A | A | A | A | A | A | A | A | A | A | A |
| S02057B | 2 | 14.5 | A | B | A | A | A | A | A | A | A | A | A | A | A | A | A | A |
| S02085 | 2 | 21.4 | A | B | A | A | A | A | A | A | A | A | A | A | A | A | A | A |
| S02085 | 2 | 21.5 | A | B | A | A | A | A | A | A | A | A | A | A | A | A | A | A |
| RM262 | 2 | 20.8 | A | B | A | A | A | A | A | A | A | A | A | A | A | A | A | A |
| RV232_SNP_nn_2 | 2 | 36.1 | A | B | A | A | A | A | A | A | B | A | A | A | A | A | A | A |
| RM166_SNP_5 | 2 | 36.2 | A | B | A | A | A | A | A | A | B | A | A | A | A | B | A | A |
| RM231_SNP_ff_1 | 3 | 2.5 | A | B | A | A | A | A | A | A | A | A | A | A | A | B | A | A |
| RM517_SNP_nn_5 | 3 | 6.3 | A | B | A | A | A | A | A | A | A | A | A | A | A | B | A | A |
| qUbi_SNP_nn_4 | 3 | 7.2 | A | B | A | A | A | A | A | A | A | A | A | A | A | B | A | A |
| S03048 | 3 | 12.1 | A | B | A | A | A | A | A | A | A | A | A | A | A | A | A | A |
| R3M30 | 3 | 20.2 | A | B | A | A | A | A | A | A | A | A | A | A | A | B | A | A |
| OsR498G0306443900_SNP_ff_1 | 3 | 23.4 | A | B | A | A | A | A | A | A | A | A | A | A | A | B | A | A |
| AGPL1_SNP_nn_2 | 3 | 33.2 | A | B | A | A | A | A | A | A | A | A | A | A | A | B | A | A |
| S03136 | 3 | 34.0 | A | B | A | A | A | A | A | A | A | A | A | A | A | A | A | A |
| RM1004_SNP_ff_2 | 3 | 35.0 | A | B | A | A | A | A | A | A | A | A | A | A | A | A | A | A |
| R4M17 | 4 | 11.8 | A | B | A | A | A | A | A | A | A | A | A | A | A | B | A | A |
| S04060 | 4 | 15.1 | A | B | A | A | A | A | A | A | A | A | A | A | A | B | A | A |
| OsR498G0408382700_SNP_ff_4 | 4 | 18.6 | A | B | A | A | A | A | A | A | A | A | A | A | A | B | A | A |
| FGR4_SNP1 | 4 | 21.6 | A | B | A | A | A | A | B | A | B | A | A | A | A | B | A | A |
| RM252_SNP_nn_4 | 4 | 25.5 | A | B | A | A | A | A | A | A | A | A | A | A | A | B | A | A |
| SPIKE_01_SNP_nn_2 | 4 | 31.6 | A | B | B | B | B | B | B | B | B | B | B | B | B | A | B | B |
| Xa2_candidate4 | 4 | 31.9 | A | B | B | A | A | A | B | B | B | B | B | B | B | B | A | B |
| S04129B | 4 | 32.4 | A | B | A | B | B | B | A | A | A | A | A | A | A | A | B | A |
| RV211_SNP_nn_2 | 4 | 34.8 | A | B | A | A | A | A | A | A | A | A | A | A | A | B | A | A |
| RM153_SNP_fn_1 | 5 | 0.2 | A | B | A | A | A | A | A | A | A | A | A | A | A | A | A | A |
| RV231_SNP_ff_1 | 5 | 0.3 | A | B | A | A | A | A | A | A | A | A | A | A | A | B | A | A |
| RV231_SNP_nn_4 | 5 | 0.3 | A | B | A | A | A | A | A | A | A | A | A | A | A | B | A | A |
| xa5S_SNP_nn_3 | 5 | 0.4 | A | B | A | A | A | A | A | A | A | A | A | A | A | B | A | A |
| xa5SR_R_SNP_nn_1 | 5 | 0.4 | A | B | A | A | A | A | A | A | A | A | A | A | A | B | A | A |
| Xa5_SNP3 | 5 | 0.4 | A | B | A | A | A | A | A | A | A | A | A | A | A | B | A | A |
| xa5_10603_T10Dw_SNP_nn_1 | 5 | 0.4 | A | B | A | A | A | A | A | A | A | A | A | A | A | B | A | A |
| Xa5_FMR_func_1 | 5 | 0.4 | A | B | A | A | A | A | A | A | A | A | A | A | A | B | A | A |
| GS5_03_1_SNP_nn_1 | 5 | 3.5 | A | B | A | A | A | A | A | A | A | A | A | A | A | B | A | A |
| RM574_SNP_nn_3 | 5 | 3.5 | A | B | A | A | A | A | A | A | A | A | A | A | A | A | A | A |
| R5M13 | 5 | 5.8 | A | B | A | A | A | A | A | A | A | A | A | A | A | A | A | A |
| OsR498G0510120000_SNP_ff_3 | 5 | 8.9 | A | B | B | B | B | B | B | B | B | B | B | B | B | B | B | B |
| R5M20 | 5 | 13.4 | A | B | A | A | A | A | A | A | A | A | A | A | A | A | A | A |
| Waxy_SNP | 6 | 1.6 | A | B | A | A | A | A | A | B | A | A | A | A | A | B | A | A |
| Amy_W2_R_1 | 6 | 1.6 | A | B | A | A | A | A | A | B | B | A | A | A | A | B | A | A |
| Amy_RM1910_func_1 | 6 | 1.6 | A | B | A | A | A | B | A | B | A | A | B | B | B | B | B | B |
| RM51_SNP_nn_2 | 7 | 0.3 | A | B | A | A | A | B | A | A | A | A | A | A | A | B | B | A |
| R7M7 | 7 | 3.5 | A | B | A | A | A | B | A | A | A | A | A | A | A | B | B | A |
| Ghd7_05_SNP_ff_1 | 7 | 9.3 | A | B | A | A | A | A | A | A | A | A | A | A | A | A | A | A |
| Xa8_NBSLRR_SNP2 | 7 | 16.3 | A | B | A | A | A | A | A | A | A | A | A | A | A | A | A | A |
| Xa8_NBSLRR_SNP3 | 7 | 16.3 | A | B | A | A | A | A | A | A | A | A | A | A | A | A | A | A |
| RM1135_SNP_nn_5 | 7 | 17.7 | A | B | A | A | A | A | A | A | A | A | A | A | A | B | A | A |
| RM10_SNP_ff_1 | 7 | 22.8 | A | B | A | A | A | A | A | A | A | A | A | A | A | B | A | A |
| R7M37 | 7 | 23.7 | A | B | A | A | A | A | A | B | B | A | A | A | A | B | A | A |
| RM7601_SNP_nn_4 | 7 | 29.6 | A | B | A | A | A | A | A | A | A | A | A | A | A | B | A | A |
| RM248_SNP_ff_2 | 7 | 29.9 | A | B | A | A | A | A | A | A | A | A | A | A | A | B | A | A |
| Os07g0695100_SNP_ff_3 | 7 | 30.2 | A | B | A | A | A | A | A | A | A | A | A | A | A | B | A | A |
| CRG4_SNP_nn_1 | 8 | 2.9 | A | B | A | A | A | A | A | A | A | A | A | A | H | A | A | A |
| Ghd8_SNP_ff_2 | 8 | 4.3 | A | B | A | A | A | A | A | A | A | A | A | A | A | B | A | A |
| RM22804 | 8 | 11.3 | A | B | A | A | A | A | A | A | A | A | A | A | H | A | A | A |
| RM7102 | 8 | 13.2 | A | B | A | A | A | A | A | A | A | A | A | A | A | A | A | A |
| RM404 | 8 | 16.5 | A | B | A | A | A | A | A | A | A | B | A | A | A | A | A | A |
| RM339_SNP_nn_5 | 8 | 19.2 | A | B | A | A | A | A | A | A | A | A | A | A | A | A | A | A |
| ESP-EAP_SNP_nn_1 | 8 | 21.7 | A | B | A | A | A | A | A | A | A | A | A | A | A | B | A | A |
| FGR8_SNP3 | 8 | 21.7 | A | B | A | A | A | A | A | A | A | A | A | A | A | B | A | A |
| badh2_E14_SNP_nn_1 | 8 | 21.8 | A | B | A | A | A | A | A | A | A | A | H | A | A | A | A | A |
| badh2_E14_SNP_nn_2 | 8 | 21.8 | A | B | A | A | A | A | A | A | A | A | A | A | A | A | A | A |
| badh2_E14_SNP_nn_3 | 8 | 21.8 | A | B | A | A | A | A | A | A | A | A | A | A | A | A | A | A |
| badh2_E14_SNP_nn_4 | 8 | 21.8 | A | B | A | A | A | A | A | A | A | A | A | A | A | A | A | A |
| RM223_SNP | 8 | 22.0 | A | B | A | A | A | A | A | A | A | A | A | A | A | B | A | A |
| RM223_SNP_nn_5 | 8 | 22.0 | A | B | A | A | A | A | A | A | A | A | A | A | A | A | A | A |
| RM80_SNP_nn_3 | 8 | 25.9 | A | B | A | A | A | A | A | A | A | A | A | A | A | A | A | A |
| xa13_prom_func_1 | 8 | 28.0 | A | B | A | A | A | A | A | A | A | A | A | A | A | A | A | A |
| xa13_prom_SNP_nn_1 | 8 | 28.0 | A | B | A | A | A | A | A | A | A | A | A | A | A | B | A | A |
| xa13_SNP_nn_2 | 8 | 28.0 | A | B | A | A | A | A | A | A | A | A | A | A | A | B | A | A |
| RM23641 | 8 | 28.3 | A | B | A | A | A | A | A | B | A | A | A | A | A | B | A | A |
| OsR498G0816560600_SNP_ff_1 | 8 | 28.6 | A | B | A | A | A | A | A | A | A | A | A | A | A | B | A | A |
| RM72 | 9 | 3.2 | A | B | A | A | A | A | A | A | A | A | A | A | A | A | A | A |
| S09040B | 9 | 10.2 | A | B | A | A | A | A | A | A | A | A | A | A | A | B | A | A |
| DEP1-1-625_SNP_nn_2 | 9 | 17.8 | A | B | A | A | A | A | A | A | A | A | A | A | A | B | A | A |
| S09073 | 9 | 18.4 | A | B | A | A | A | A | A | A | A | A | A | A | A | A | A | A |
| RM288_SNP_ff_1 | 9 | 20.0 | A | B | A | A | A | A | A | A | A | A | A | A | A | A | A | A |
| RM242_SNP_nn_4 | 9 | 20.3 | A | B | A | A | A | A | A | A | A | A | A | A | A | A | A | A |
| RM201_SNP_nn_5 | 9 | 21.7 | A | B | A | A | A | A | A | A | A | A | A | A | A | A | A | A |
| S10001 | 10 | 0.5 | A | B | A | A | A | A | A | A | A | A | A | A | A | A | A | A |
| S10003A | 10 | 0.8 | A | B | A | A | A | A | A | A | A | A | A | A | A | A | A | A |
| RM271 | 10 | 3.5 | A | B | A | A | A | A | A | A | A | A | A | A | A | A | A | A |
| RM216 | 10 | 4.6 | A | B | A | A | A | A | A | A | A | A | B | A | A | A | A | A |
| OsR498G1018206100_SNP_ff_1 | 10 | 4.7 | A | B | A | A | A | A | A | A | A | A | B | A | A | B | A | A |
| R10M17 | 10 | 9.0 | A | B | A | A | A | A | A | A | A | A | A | A | A | A | A | A |
| R10M30 | 10 | 17.0 | A | B | A | A | A | A | A | A | A | A | A | A | A | A | A | A |
| R10M40 | 10 | 19.5 | A | B | A | A | A | A | A | A | A | A | A | A | A | A | A | A |
| RM171_SNP_nn_1 | 10 | 21.3 | A | B | A | A | A | A | A | A | A | A | B | A | A | B | A | A |
| RM590_SNP_ff_1 | 10 | 22.0 | A | B | A | A | A | A | A | A | A | A | A | A | A | B | A | A |
| HVSSR11-12 | 11 | 2.5 | A | B | A | A | A | A | A | A | A | A | A | A | A | A | A | A |
| RM202 | 11 | 7.5 | A | B | A | A | A | A | A | A | A | A | A | A | A | A | A | A |
| RM26746 | 11 | 16.8 | A | B | B | A | B | A | A | A | A | A | A | A | A | A | A | A |
| R11M23 | 11 | 19.2 | A | B | B | H | B | B | B | B | B | B | B | B | B | B | B | B |
| RM27000 | 11 | 21.8 | A | B | B | B | B | B | B | B | B | B | B | B | B | B | B | B |
| Pi54_MAS_SNP_nf_1 | 11 | 22.0 | A | B | B | B | B | B | B | B | B | B | B | B | B | B | B | B |
| C189-Xa23_SNP_nn_1 | 11 | 24.5 | A | B | B | B | B | B | B | B | B | B | B | B | B | B | B | B |
| CKM -1 | 11 | 27.5 | A | B | B | B | B | B | B | B | B | B | B | B | B | B | B | B |
| Pik_h_SNP_nn_4 | 11 | 27.9 | A | B | B | B | B | B | B | B | B | B | B | B | B | B | B | B |
| RM144 | 11 | 28.2 | A | B | H | H | A | A | A | A | A | A | A | A | A | A | A | A |
| RM415 | 12 | 1.0 | A | B | A | A | A | A | A | A | A | A | A | A | A | A | A | A |
| RM453 | 12 | 2.6 | A | B | A | A | A | A | A | A | A | A | A | A | A | A | A | A |
| RM7619 | 12 | 4.8 | A | B | A | A | A | A | A | A | A | A | A | A | A | A | A | A |
| RM44 | 12 | 8.9 | A | B | B | B | B | B | B | B | B | B | B | B | B | B | B | B |
| Pita_Pita_2_b_SNP_nn_1 | 12 | 9.7 | A | B | A | A | A | A | A | A | A | A | A | A | A | A | A | A |
| Pita_Pita_2_b_SNP_nf_1 | 12 | 9.7 | A | B | A | A | A | A | A | A | A | A | A | A | A | A | A | A |
| YL153_YL154func_2 | 12 | 9.7 | A | B | A | A | A | A | A | A | A | A | A | A | A | A | A | A |
| RM27933 | 12 | 10.4 | A | B | B | B | B | B | B | B | B | B | B | B | B | B | B | B |
| RM27966 | 12 | 12.2 | A | B | B | B | B | B | B | B | B | B | B | B | B | B | B | B |
| RM7102_SNP2 | 12 | 12.5 | A | B | B | B | B | B | B | B | B | B | B | B | B | B | B | B |
| RM27462 | 12 | 14.1 | A | B | A | A | A | A | A | A | A | A | A | A | A | A | A | A |
| RM19 | 12 | 14.3 | A | B | A | A | A | A | A | H | A | A | A | A | A | A | A | A |
| PBA14 | 12 | 10.4 | A | B | A | A | A | A | A | A | A | A | A | A | A | A | A | A |
| OSM89 | 12 | 11.3 | A | B | A | A | A | H | A | A | A | A | A | A | A | A | A | A |

**Table S5. Foreground selection of Inter-cross F_2_ population carrying blast resistance genes *Pi9+Pi54* in the background of *Mushk Budji***

| **IC-BC2F1 Parent#ID** | **BC2F2#ID** | ***Pi54*** | ***Pi9*** |  | **PH (cm)** | **ET** | **PL (cm)** | **SP** | **SF (%)** | **GY**  **(g)** |
| --- | --- | --- | --- | --- | --- | --- | --- | --- | --- | --- |
| 1. SKUA-528-50-1-1- 3-2 | 1 | B | H |  | 119 | 21 | 25 | 185 | 93.4 | 24.6 |
| 1. SKUA-528-50-1-1- 3-2 | 4 | H | B |  | 95 | 16 | 19 | 160 | 77.2 | 13.4 |
| 1. SKUA-528-50-1-1- 3-2 | 18 | **B** | **B** |  | 135 | 17 | 17 | 86 | 63.1 | 11.6 |
| 1. SKUA-528-50-1-1- 3-2 | 19 | **B** | **B** |  | 101 | 17 | 19 | 116 | 60.4 | 12.0 |
| 1. SKUA-528-50-1-1- 3-2 | 23 | B | H |  | 116 | 19 | 18 | 94 | 65.1 | 12.2 |
| 1. SKUA-528-50-1-1- 19-1 | 1 | H | B |  | 112 | 17 | 22 | 113 | 70.0 | 18.3 |
| 1. SKUA-528-50-1-1- 19-1 | 2 | H | A |  | 117 | 14 | 18 | 108 | 79.7 | 16.8 |
| 1. SKUA-528-50-1-1- 19-1 | 3 | H | B |  | 110 | 15 | 21 | 97 | 67.8 | 19.5 |
| 1. SKUA-528-50-1-1- 19-1 | 5 | **B** | **B** |  | 110 | 17 | 20 | 126 | 74.2 | 19.1 |
| 1. SKUA-528-50-1-1- 19-1 | 6 | B | A |  | 101 | 20 | 17 | 126 | 75.7 | 19.5 |
| 1. SKUA-528-50-1-1- 19-1 | 8 | H | B |  | 109 | 17 | 17 | 97 | 65.5 | 14.0 |
| 1. SKUA-528-50-1-1- 19-1 | 11 | B | H |  | 130 | 19 | 18 | 168 | 85.0 | 11.3 |
| 1. SKUA-528-50-1-1- 19-1 | 15 | B | H |  | 110 | 20 | 17 | 122 | 72.0 | 10.3 |
| 1. SKUA-528-50-1-1- 19-1 | 17 | H | B |  | 101 | 16 | 19 | 165 | 67.3 | 19.6 |
| 1. SKUA-528-50-1-1- 19-1 | 18 | H | H |  | 106 | 20 | 18 | 96 | 67.5 | 10.8 |
| 1. SKUA-528-50-1-1- 19-1 | 20 | B | A |  | 113 | 19 | 21 | 119 | 79.9 | 17.0 |
| 1. SKUA-528-50-1-1- 19-1 | 34 | H | B |  | 130 | 21 | 22 | 119 | 82.9 | 11.6 |
| 1. SKUA-528-50-1-1- 19-1 | 25 | H | A |  | 107 | 14 | 21 | 132 | 61.9 | 16.7 |
| 1. SKUA-528-50-1-1- 19-1 | 28 | A | B |  | 126 | 19 | 20 | 117 | 78.6 | 14.1 |
| 1. SKUA-528-50-1-1- 19-1 | 29 | H | H |  | 100 | 21 | 17 | 102 | 79.4 | 18.0 |
| 1. SKUA-528-50-1-1- 19-1 | 30 | B | H |  | 130 | 17 | 17 | 166 | 60.6 | 17.6 |
| 1. SKUA-528-50-1-1- 19-1 | 32 | H | H |  | 115 | 20 | 22 | 115 | 65.9 | 11.0 |
| 1. SKUA-528-50-1-1- 19-1 | 37 | **B** | **B** |  | 109 | 13 | 22 | 169 | 80.1 | 11.3 |
| 1. SKUA-528-50-1-1- 19-1 | 38 | B | H |  | 130 | 22 | 18 | 114 | 62.2 | 13.4 |
| 1. SKUA-528-50-1-1- 19-1 | 39 | **B** | **B** |  | 109 | 17 | 18 | 170 | 78.1 | 14.5 |
| 1. SKUA-528-50-1-1- 19-1 | 40 | H | H |  | 125 | 18 | 21 | 163 | 85.9 | 19.8 |
| 1. SKUA-528-50-1-1- 19-1 | 45 | H | B |  | 107 | 18 | 17 | 150 | 83.5 | 10.2 |
| 1. SKUA-528-50-1-1- 19-1 | 47 | **B** | **B** |  | 132 | 20 | 23 | 87 | 61.3 | 15.0 |
| 1. SKUA-528-50-1-1- 19-1 | 50 | A | H |  | 114 | 18 | 22 | 117 | 73.2 | 13.0 |
| 1. SKUA-528-50-1-1- 19-1 | 74 | H | B |  | 103 | 13 | 17 | 85 | 63.6 | 19.7 |
| 1. SKUA-528-50-1-1- 19-1 | 75 | **B** | **B** |  | 98 | 13 | 21 | 133 | 68.4 | 18.6 |
| 1. SKUA-528-50-1-1- 19-1 | 78 | H | H |  | 103 | 15 | 17 | 104 | 74.7 | 12.5 |
| 1. SKUA-528-50-1-1- 19-1 | 80 | B | H |  | 113 | 15 | 17 | 161 | 63.8 | 16.3 |
| 1. SKUA-528-50-1-1- 19-1 | 81 | B | H |  | 101 | 22 | 22 | 118 | 61.2 | 15.8 |
| 1. SKUA-528-50-1-1- 19-1 | 83 | H | B |  | 123 | 21 | 21 | 93 | 61.1 | 16.9 |
| 1. SKUA-528-50-1-1- 19-1 | 85 | H | B |  | 113 | 13 | 22 | 104 | 61.0 | 16.1 |
| 1. SKUA-528-50-1-1- 19-1 | 86 | H | A |  | 98 | 20 | 22 | 122 | 81.3 | 12.4 |
| 1. SKUA-528-50-1-1- 19-1 | 90 | H | H |  | 98 | 21 | 18 | 103 | 85.0 | 12.1 |
| 1. SKUA-528-50-1-1- 19-1 | 93 | H | B |  | 131 | 17 | 22 | 118 | 63.4 | 12.5 |
| 1. SKUA-528-50-1-1- 19-1 | 94 | **B** | **B** |  | 134 | 20 | 17 | 125 | 76.2 | 17.4 |
| 1. SKUA-528-50-1-1- 19-1 | 100 | B | H |  | 121 | 20 | 18 | 135 | 62.0 | 17.1 |
| 1. SKUA-528-50-1-1- 19-1 | 103 | H | H |  | 134 | 20 | 19 | 109 | 64.1 | 10.7 |
| 1. SKUA-528-50-1-1- 19-5 | 1 | H | A |  | 101 | 22 | 20 | 105 | 73.9 | 17.0 |
| 1. SKUA-528-50-1-1- 19-5 | 2 | A | H |  | 107 | 17 | 19 | 142 | 73.9 | 10.9 |
| 1. SKUA-528-50-1-1- 19-5 | 3 | A | B |  | 99 | 21 | 23 | 145 | 61.6 | 16.2 |
| 1. SKUA-528-50-1-1- 19-5 | 4 | A | A |  | 131 | 17 | 22 | 125 | 67.3 | 14.2 |
| 1. SKUA-528-50-1-1- 19-5 | 5 | A | H |  | 118 | 20 | 22 | 169 | 85.2 | 10.2 |
| 1. SKUA-528-50-1-1- 19-5 | 6 | A | B |  | 125 | 14 | 17 | 123 | 70.2 | 18.9 |
| 1. SKUA-528-50-1-1- 19-5 | 7 | A | H |  | 101 | 22 | 19 | 109 | 66.2 | 16.7 |
| 1. SKUA-528-50-1-1- 19-5 | 8 | A | B |  | 122 | 15 | 17 | 90 | 66.8 | 12.4 |
| 1. SKUA-528-50-1-1- 19-5 | 9 | A | B |  | 133 | 16 | 20 | 88 | 63.6 | 13.3 |
| 1. SKUA-528-50-1-1- 19-5 | 10 | A | H |  | 98 | 16 | 22 | 151 | 70.0 | 12.3 |
| 1. SKUA-528-50-1-1- 19-5 | 11 | A | A |  | 121 | 17 | 19 | 121 | 77.5 | 18.7 |
| 1. SKUA-528-50-1-1- 19-5 | 12 | A | A |  | 99 | 13 | 20 | 81 | 80.4 | 18.4 |
| PH (cm): Plant height, ET: Number of effective tillers per plant, PL (cm): Panicle length, SP: Spikelets per panicle, SF (%): Spikelet fertility, GY (g): Grain yield per plant; Shaded rows depict the selected plants based on marker genotype; A: recipient parent allele, B: Donor allele (linked to target gene); H: heterozygous; Bold figures indicate double homozygotes for B allele | | | | | | | | | | |

**Table S6. Foreground selection of Inter-cross F_2_ population carrying blast resistance gene *Pi9* in the background of *Mushk Budji***

| **IC-BC_2_F_1_ Parent#ID** | **IC-BC_2_F_2_#ID** | **Pi9** |  | **PH (cm)** | **ET** | **PL (cm)** | **SP** | **SF (%)** | **GY(g)** |
| --- | --- | --- | --- | --- | --- | --- | --- | --- | --- |
| 1. SKUA-528-50-1-1- 19-6 | 1 | **B** |  | 132 | 18 | 23 | 131 | 64.8 | 13.2 |
| 1. SKUA-528-50-1-1- 19-6 | 2 | **B** |  | 106 | 14 | 18 | 136 | 76.3 | 11.9 |
| 1. SKUA-528-50-1-1- 19-6 | 3 | **B** |  | 95 | 20 | 17 | 123 | 78.6 | 19.7 |
| 1. SKUA-528-50-1-1- 3-1 | 1 | A |  | 105 | 19 | 23 | 93 | 71.2 | 10.1 |
| 1. SKUA-528-50-1-1- 3-1 | 2 | A |  | 125 | 19 | 18 | 167 | 71.6 | 17.0 |
| 1. SKUA-528-50-1-1- 3-1 | 3 | A |  | 108 | 17 | 22 | 160 | 61.3 | 16.2 |
| 1. SKUA-528-50-1-1- 3-1 | 4 | A |  | 119 | 14 | 20 | 120 | 64.5 | 11.7 |
| 1. SKUA-528-50-1-1- 3-1 | 5 | A |  | 111 | 18 | 20 | 127 | 61.1 | 16.2 |
| 1. SKUA-528-50-1-1- 3-1 | 6 | A |  | 110 | 20 | 19 | 140 | 67.6 | 17.6 |
| 1. SKUA-528-50-1-1- 3-1 | 7 | A |  | 100 | 18 | 17 | 80 | 74.9 | 10.3 |
| 1. SKUA-528-50-1-1- 3-1 | 8 | A |  | 119 | 21 | 23 | 112 | 62.1 | 16.2 |
| 1. SKUA-528-50-1-1- 3-1 | 9 | A |  | 117 | 20 | 21 | 105 | 66.0 | 16.3 |
| 1. SKUA-528-50-1-1- 3-1 | 10 | A |  | 128 | 14 | 23 | 158 | 63.0 | 19.9 |
| 1. SKUA-528-50-1-1- 3-1 | 11 | A |  | 100 | 14 | 17 | 146 | 77.9 | 18.3 |
| 1. SKUA-528-50-1-1- 3-1 | 12 | A |  | 134 | 13 | 21 | 157 | 79.7 | 15.3 |
| 1. SKUA-528-50-1-1- 3-1 | 13 | A |  | 121 | 20 | 18 | 134 | 69.0 | 18.5 |
| 1. SKUA-528-50-1-1- 3-1 | 14 | A |  | 103 | 18 | 17 | 95 | 74.8 | 11.7 |
| 1. SKUA-528-50-1-1- 3-1 | 15 | A |  | 107 | 22 | 17 | 109 | 80.7 | 10.0 |
| 1. SKUA-528-50-1-1- 3-1 | 16 | A |  | 133 | 15 | 18 | 141 | 72.6 | 15.9 |
| 1. SKUA-528-50-1-1- 19-2 | 5 | H |  | 102 | 16 | 18 | 88 | 74.7 | 16.9 |
| 1. SKUA-528-50-1-1- 19-2 | 13 | **B** |  | 123 | 13 | 20 | 158 | 74.4 | 19.1 |
| 1. SKUA-528-50-1-1- 19-2 | 38 | H |  | 94 | 14 | 18 | 98 | 71.1 | 18.1 |
| 1. SKUA-528-50-1-1- 19-2 | 50 | H |  | 126 | 18 | 22 | 115 | 66.7 | 16.8 |
| **IRBL 9W** |  |  |  | **117.8** | **10.9** | **14.7** | **128.7** | **82.7** | **17.3** |
| **DHMAS 70Q 164-1b** |  |  |  | **105.6** | **8.7** | **16.5** | **108.8** | **56.3** | **12.8** |
| **Mushk Budji** |  |  |  | **126.8** | **17.4** | **19.9** | **135.1** | **85.2** | **23.4** |

PH (cm): Plant height, ET: Number of effective tillers per plant, PL (cm): Panicle length, SP: Spikelets per panicle, SF (%): Spikelet fertility, GY (g): Grain yield per plant; Shaded rows depict the selected plants based on marker genotype; A: recipient parent allele, B: Donor allele (linked to target gene); H: heterozygous

**Table S7. Foreground selection of Inter-cross F_2_ population carrying blast resistance gene *Pi54* in the background of *Mushk Budji***

| **IC-BC_2_F_1_ Parent#ID** | **IC-BC_2_F_2_#ID** | **Pi54** |  | **PH (cm)** | **ET** | **PL (cm)** | **SP** | **SF (%)** | **GY (g)** |
| --- | --- | --- | --- | --- | --- | --- | --- | --- | --- |
| 1. SKUA-528-50-1-1- 3-2 | 1 | **B** |  | 129 | 21 | 25 | 185 | 93.8 | 25.0 |
| 1. SKUA-528-50-1-1- 3-2 | 2 | **B** |  | 120 | 13 | 17 | 148 | 79.3 | 12.2 |
| 1. SKUA-528-50-1-1- 3-2 | 3 | H |  | 101 | 21 | 25 | 185 | 93.3 | 25.0 |
| 1. SKUA-528-50-1-1- 3-2 | 4 | **B** |  | 117 | 13 | 21 | 168 | 73.7 | 19.8 |
| 1. SKUA-528-50-1-1- 3-2 | 5 | **B** |  | 111 | 21 | 25 | 185 | 93.7 | 25.2 |
| 1. SKUA-528-50-1-1- 3-2 | 6 | H |  | 128 | 19 | 19 | 149 | 63.1 | 18.2 |
| 1. SKUA-528-50-1-1- 3-2 | 7 | H |  | 119 | 21 | 25 | 185 | 93.9 | 24.4 |
| 1. SKUA-528-50-1-1- 3-2 | 8 | **B** |  | 111 | 18 | 23 | 125 | 71.4 | 18.3 |
| 1. SKUA-528-50-1-1- 3-2 | 9 | A |  | 125 | 21 | 25 | 185 | 93.5 | 25.0 |
| 1. SKUA-528-50-1-1- 3-2 | 10 | **B** |  | 120 | 19 | 22 | 93 | 72.1 | 13.3 |
| 1. SKUA-528-50-1-1- 3-2 | 11 | H |  | 132 | 21 | 25 | 185 | 93.4 | 24.6 |
| 1. SKUA-528-50-1-1- 3-2 | 12 | **B** |  | 131 | 16 | 22 | 94 | 81.0 | 19.9 |
| 1. SKUA-528-50-1-1- 3-2 | 13 | **B** |  | 122 | 21 | 25 | 185 | 93.3 | 25.2 |
| 1. SKUA-528-50-1-1- 3-2 | 14 | **B** |  | 117 | 16 | 19 | 123 | 80.5 | 16.4 |
| 1. SKUA-528-50-1-1- 3-2 | 15 | H |  | 120 | 21 | 25 | 185 | 93.5 | 24.5 |
| 1. SKUA-528-50-1-1- 3-2 | 19 | A |  | 121 | 22 | 23 | 153 | 63.6 | 11.5 |

PH (cm): Plant height, ET: Number of effective tillers per plant, PL (cm): Panicle length, SP: Spikelets per panicle, SF (%): Spikelet fertility, GY (g): Grain yield per plant; Shaded rows depict the selected plants based on marker genotype; A: recipient parent allele, B: Donor allele (linked to target gene); H: heterozygous
