# Supplementary material for: Genomics-informed elucidation of trait-phenotype relationships and MABB approaches deliver major gene blast resistance in the aromatic rice landrace Mushk Budji
Source: Front Genet. 2026 Jan 22;16:1699333. doi: 10.3389/fgene.2025.1699333 (PMC12872327; doi:10.3389/fgene.2025.1699333)

**Figure S1. SNP marker distribution on carrier and non-carrier chromosomes used for estimation of genome recovery of pyramided lines and NILs**


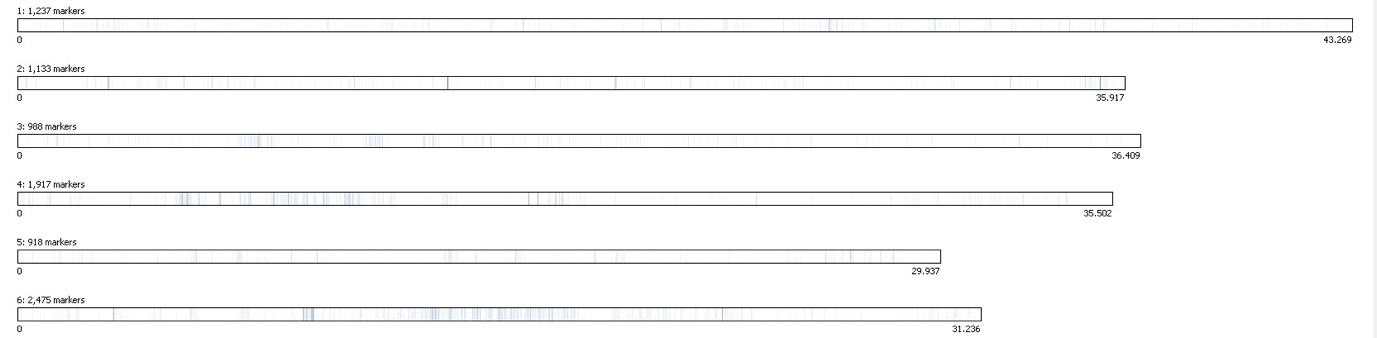

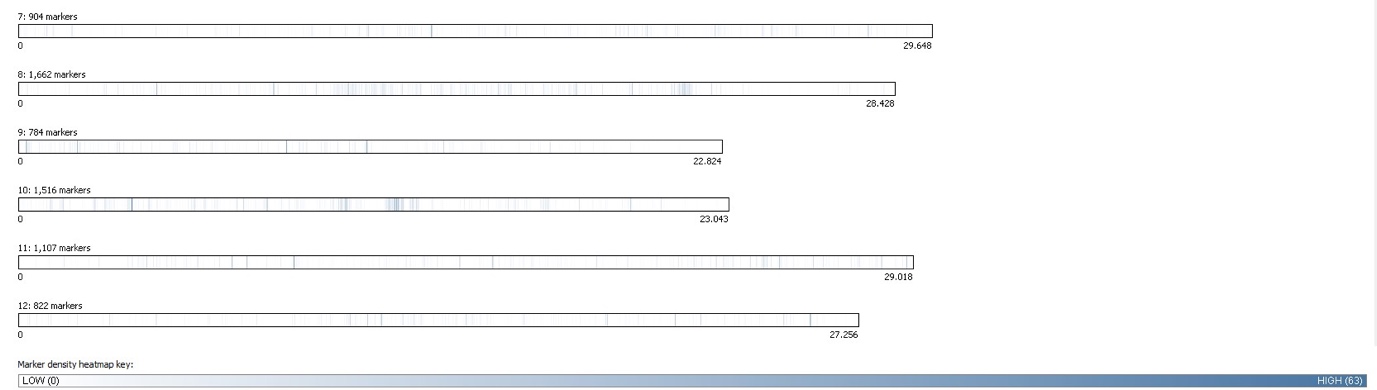


**Figure S2. Graphical genotype of non-carrier chromosomes depicting genome similarity of pyramided lines and NILs towards the recurrent parent, *Mushk Budji***


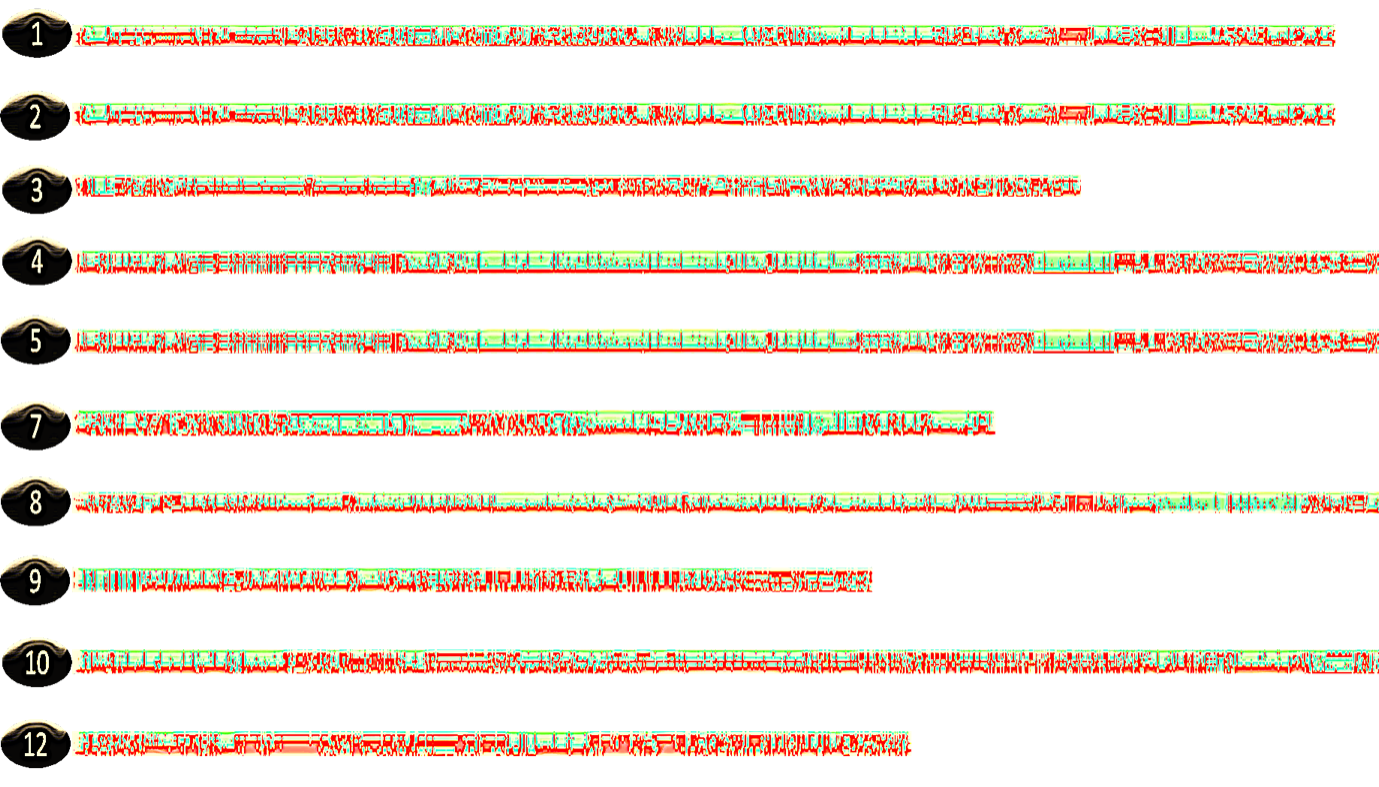


**Figure S3 Sequence level validation of pyramided lines for important genes related to early flowering cold tolerance and grain traits**

***Alk***
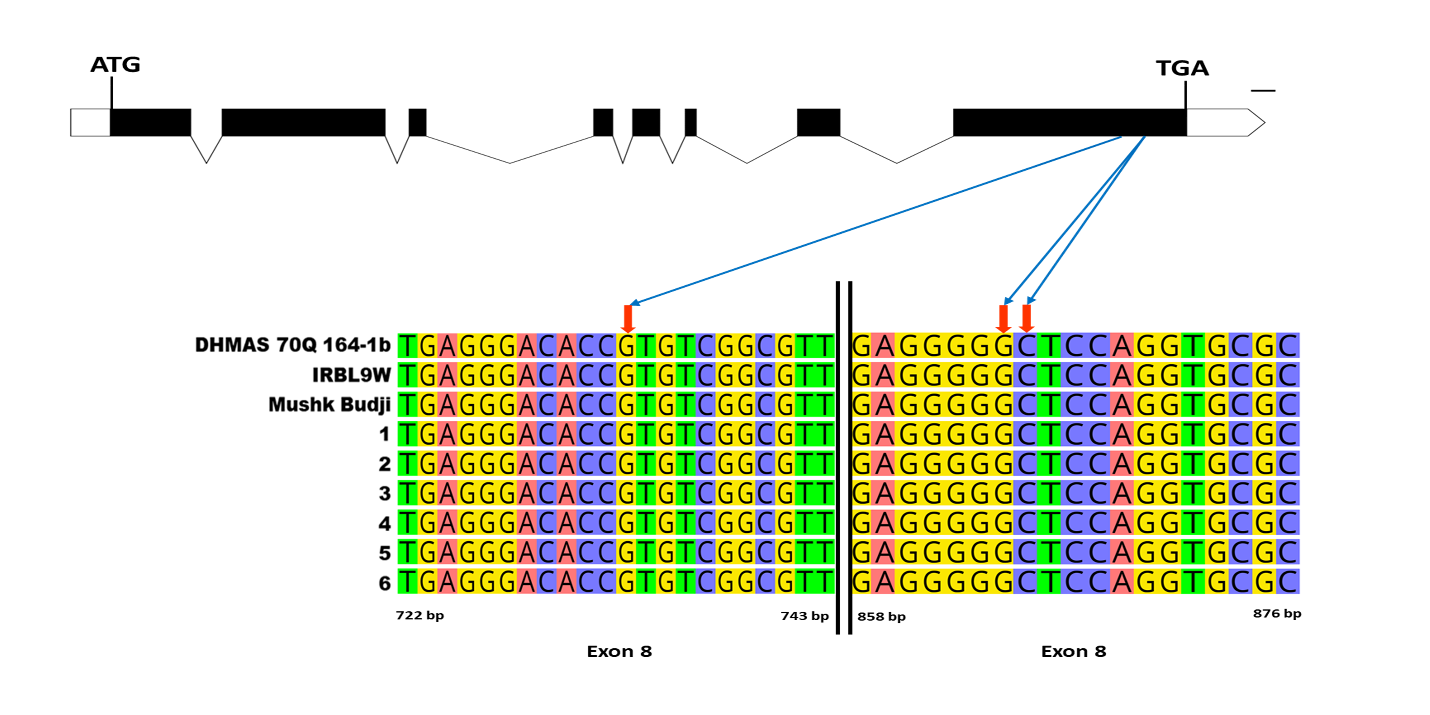


***Hd4***

***
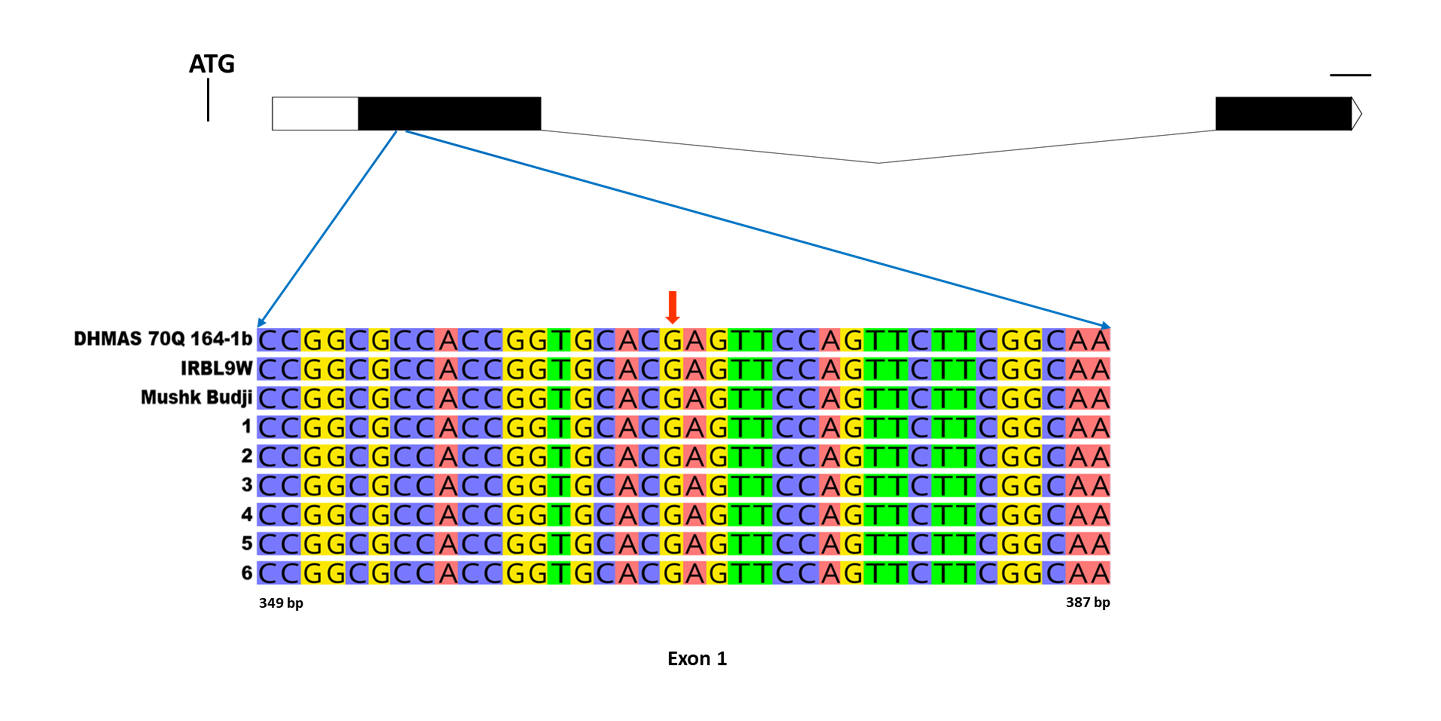
***

***Hd5***

***
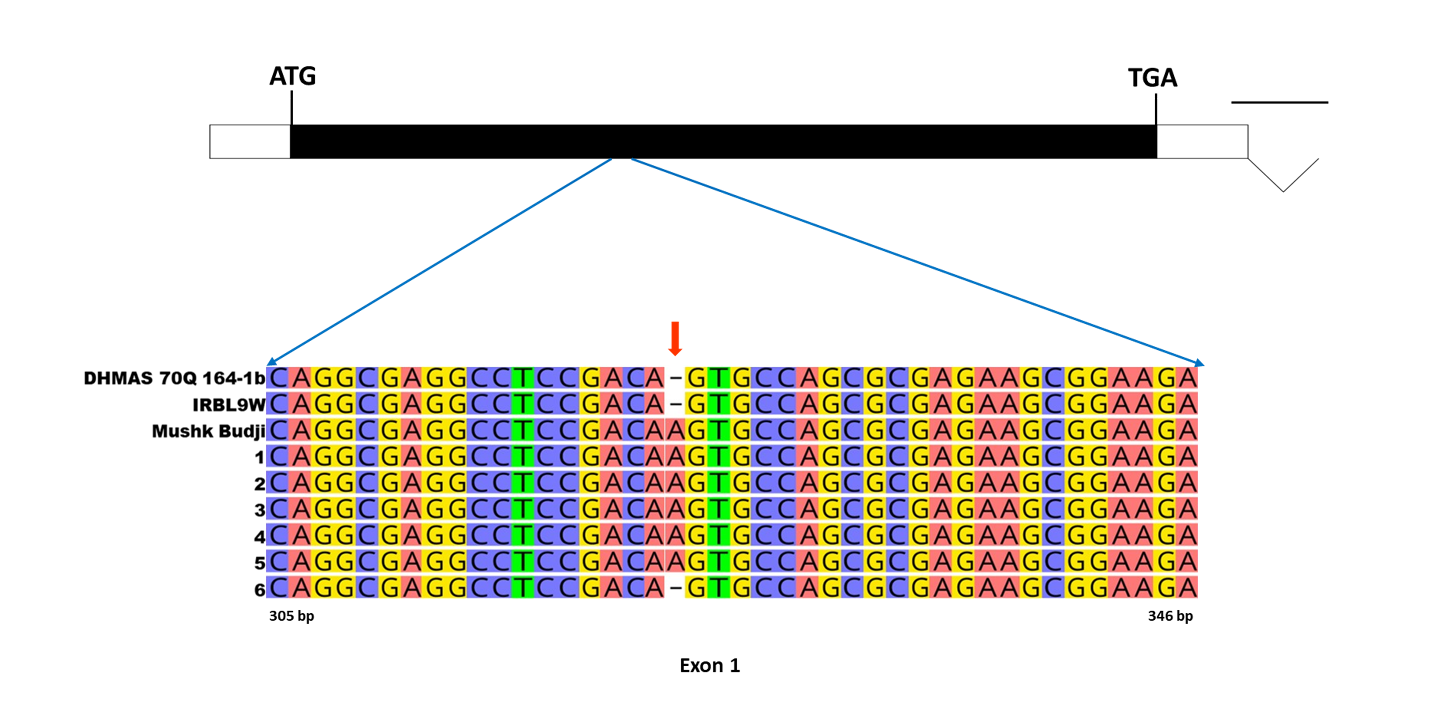
***

***Cold1***

***
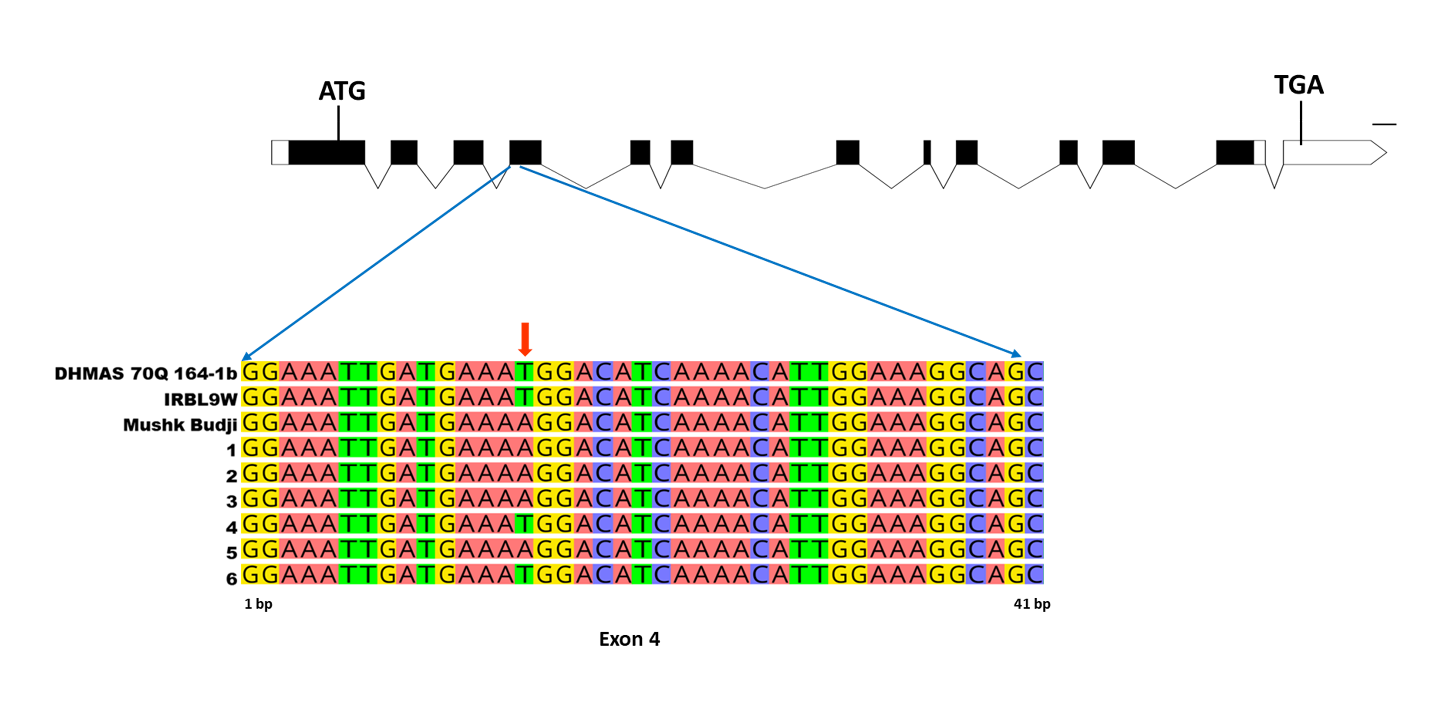
***

***Cold6***

***
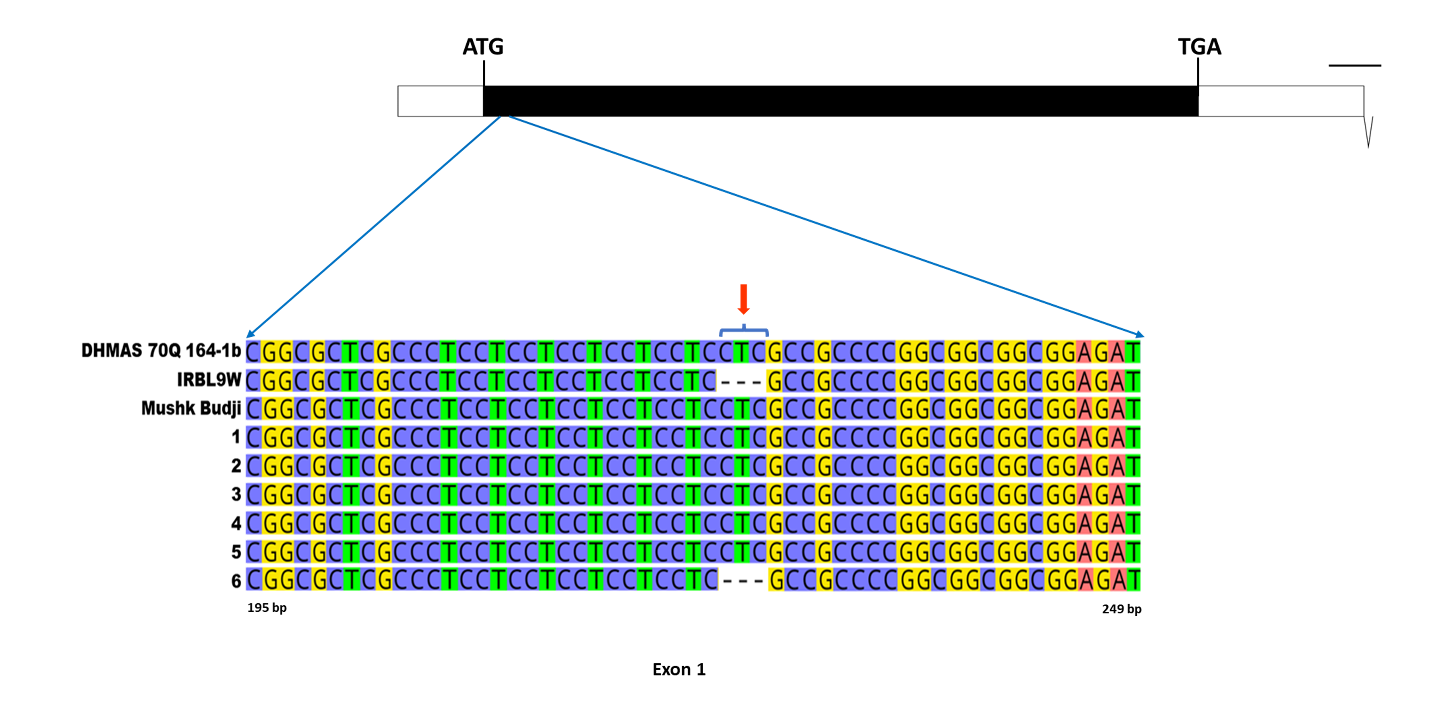
***

***LABA1***

***
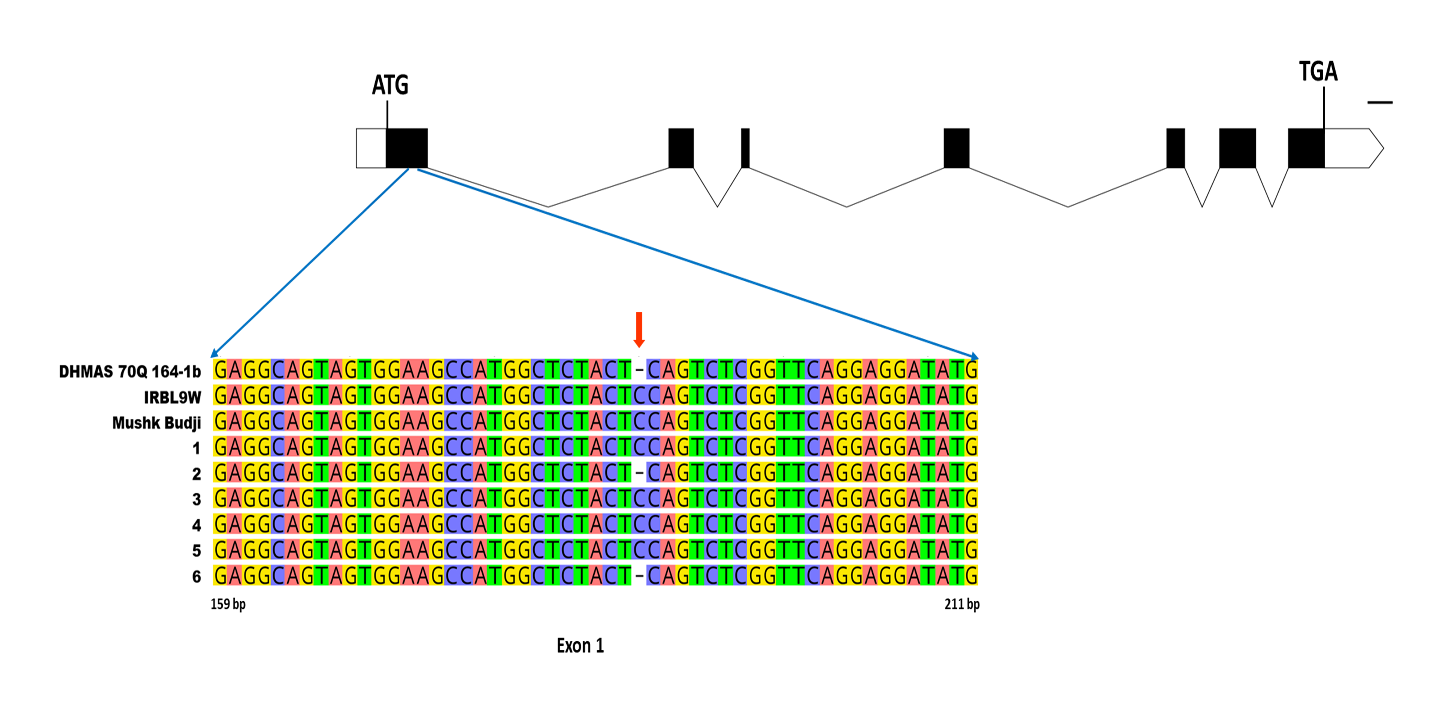
***

***RC***

***
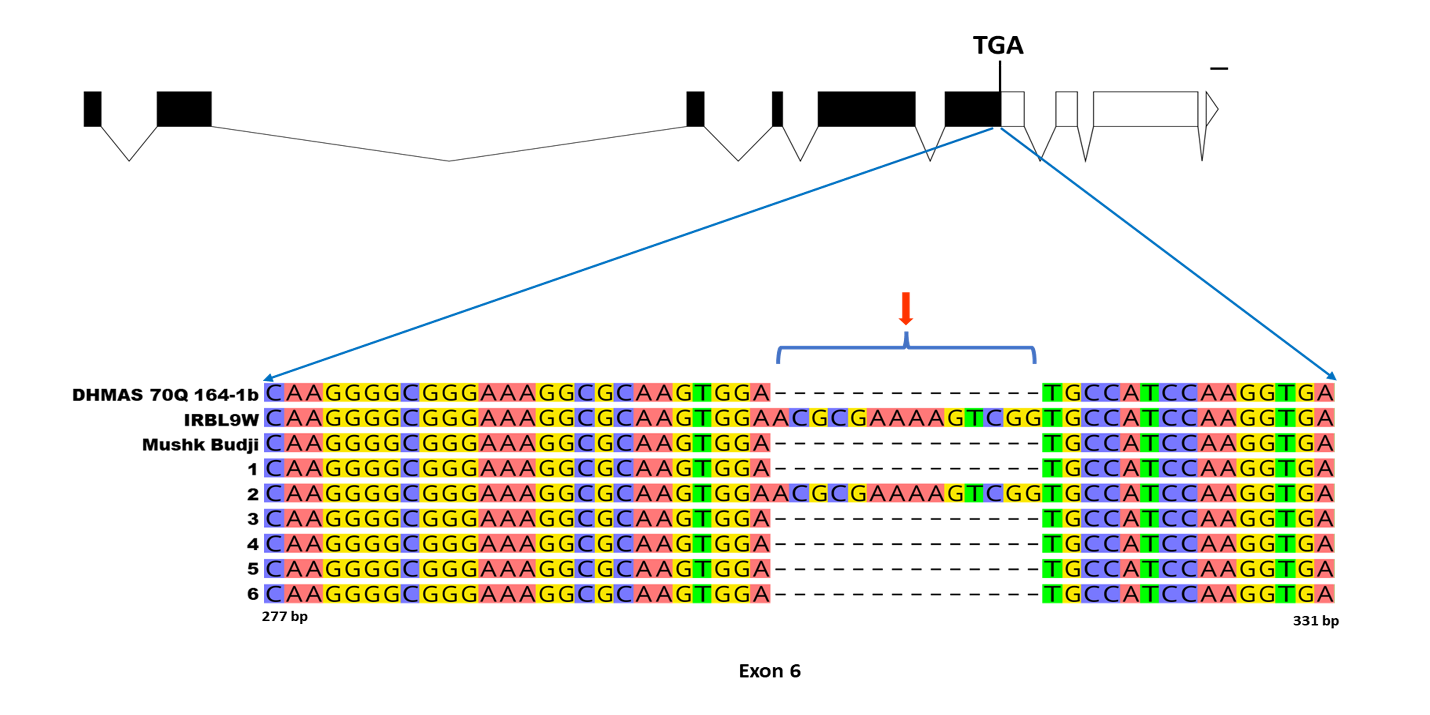
***

**DHMAS 70Q 164-1b; IRBL9W; Mushk Budji; 1: SKUA-528-50-1-1-19-1-94; 2: SKUA-528-50-1-1-19-2-13; 3: SKUA-528-50-1-1-19-1-5; 4: SKUA-528-50-1-1-3-2-18; 5: SKUA-528-50-1-1-19-1-37; 6: SKUA-528-50-1-1-3-2-8**

**Figure S4. Multi-location testing of gene pyramided lines at farmers field (Location: J&K, India)**


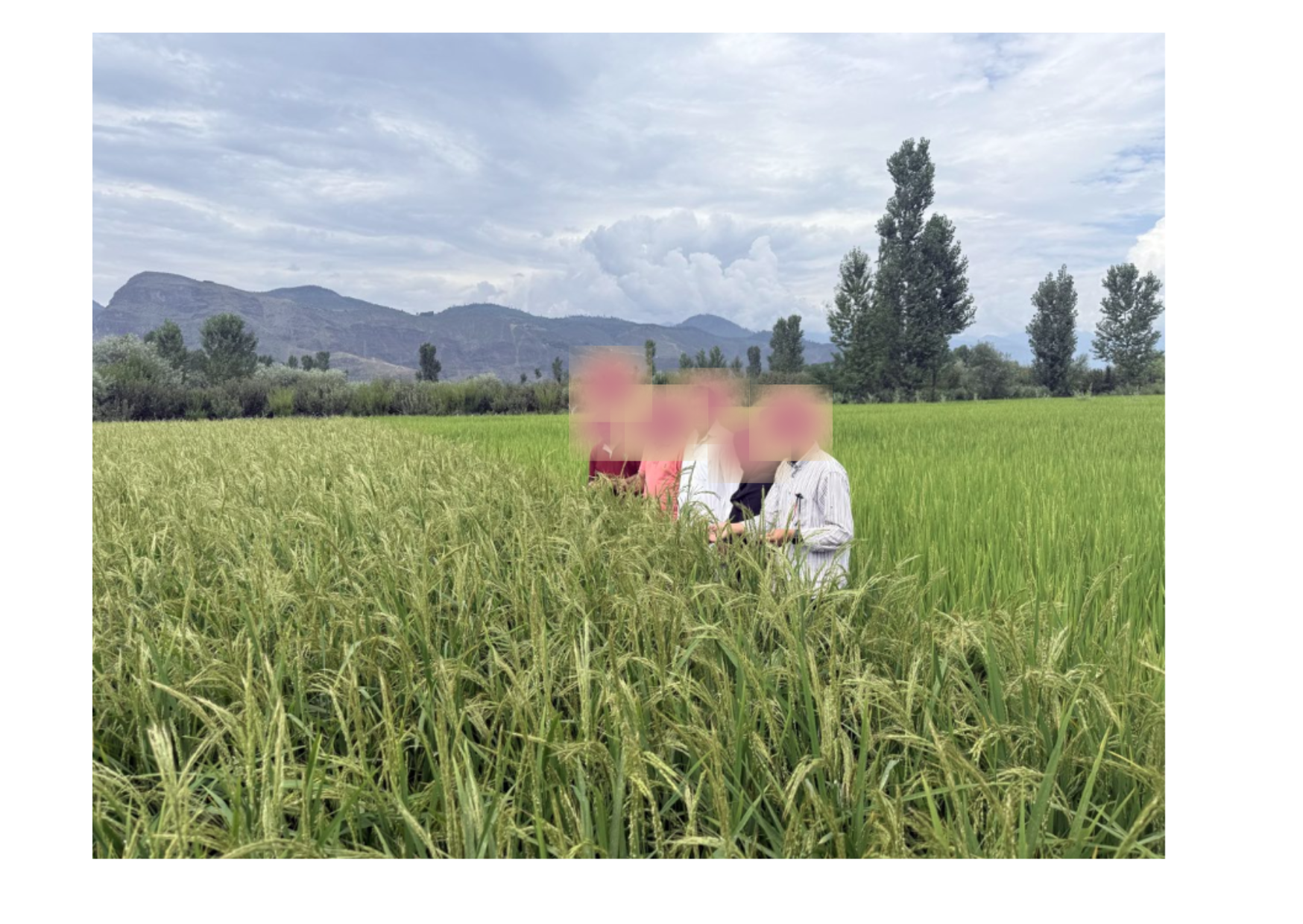

Supplement: Supplementary file 3 [file DataSheet1.docx]
